# The recombinant shingles vaccine is associated with lower risk of dementia

**DOI:** 10.1038/s41591-024-03201-5

**Published:** 2024-07-25

**Authors:** Maxime Taquet, Quentin Dercon, John A. Todd, Paul J. Harrison

**Affiliations:** 1 Department of Psychiatry, University of Oxford, Warneford Hospital, Oxford, UK; 2 Oxford Health NHS Foundation Trust, Warneford Hospital, Oxford, UK; 3 Max Planck Centre for Computational Psychiatry and Ageing Research, Queen Square Institute of Neurology and Mental Health Neuroscience Department, Division of Psychiatry, University College London, London, UK; 4 Centre for Human Genetics, Nuffield Department of Medicine, University of Oxford, Oxford, UK

**Keywords:** Dementia, Viral infection

## Abstract

There is emerging evidence that the live herpes zoster (shingles) vaccine might protect against dementia. However, the existing data are limited and refer only to the live vaccine, which is now discontinued in the United States and many other countries in favor of a recombinant vaccine. Whether the recombinant shingles vaccine protects against dementia remains unknown. Here we used a natural experiment opportunity created by the rapid transition from the use of live to the use of recombinant vaccines to compare the risk of dementia between vaccine types. We show that the recombinant vaccine is associated with a significantly lower risk of dementia in the 6 years post-vaccination. Specifically, receiving the recombinant vaccine is associated with a 17% increase in diagnosis-free time, translating into 164 additional days lived without a diagnosis of dementia in those subsequently affected. The recombinant shingles vaccine was also associated with lower risks of dementia than were two other vaccines commonly used in older people: influenza and tetanus–diphtheria–pertussis vaccines. The effect was robust across multiple secondary analyses, and was present in both men and women but was greater in women. These findings should stimulate studies investigating the mechanisms underpinning the protection and could facilitate the design of a large-scale randomized control trial to confirm the possible additional benefit of the recombinant shingles vaccine.

## Abstract (web summary)

A natural experiment including more than 200,000 people who received a shingles vaccine reveals that, within 6 years of vaccination, the recombinant vaccine is associated with lower risk of dementia than is the live vaccine.

## Main

Varicella-zoster virus is a herpesvirus that causes chickenpox (varicella) and shingles (herpes zoster). Given the risk of deleterious consequences of shingles^1^, in many countries vaccination is now recommended for older adults. Recent studies have generated substantial interest in the potential protective effect of shingles vaccination against dementia^2–7^. However, most have compared vaccinated with unvaccinated cohorts, a design prone to selection bias, including healthy-vaccinee bias, meaning that individuals who decide to get vaccinated are generally healthier than those who choose not to^8^. The only exception is a recent natural experiment that compared people just above and just below the eligibility age cut-off: this study provided evidence that live shingles vaccination might protect against dementia^3^. The effect was present only in women and was limited to the live vaccine, which is now discontinued in the United States and is being withdrawn in many other countries, in favor of a recombinant vaccine. Whether the latter provides protection against dementia remains unknown^7^.

**Fig. 1:**
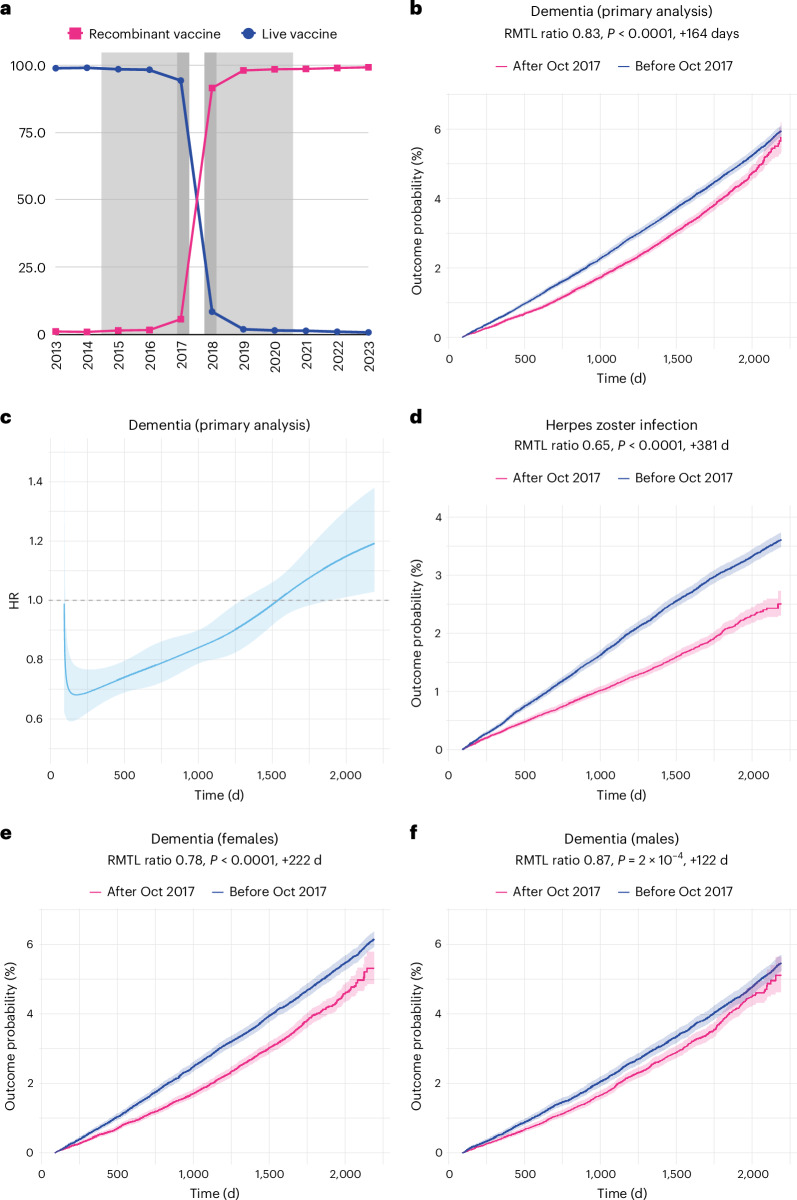
Association between recombinant shingles vaccine and risk of dementia within 6 years of vaccination. **a**, Proportion (in %) of each vaccine being received, showing the step change that occurred in October 2017. The exposure windows used in the primary analysis are shown in gray, and the restricted exposure windows that were used in a robustness analysis are shown in dark gray. **b**, Curves representing the Kaplan–Meier estimates of the cumulative incidence of dementia diagnosis in the 3 months to 6 years after shingles vaccination in the primary analysis (*n* = 103,837 in each cohort). **c**, Curve representing the time-varying hazard ratio (HR) for the risk of dementia in the primary analysis (HR < 1 indicates a lower risk of dementia in those who received the vaccine after October 2017); *n* = 103,837 in each cohort. **d**, Curves representing the Kaplan–Meier estimates of the cumulative incidence for herpes zoster infection (*n* = 103,837 in each cohort). **e**,**f**, Curves representing the Kaplan–Meier estimates of the cumulative incidence of dementia among women (**e**) and men (**f**) (*n* = 54,846 in each cohort for females, and *n* = 43,990 for males). The RMTL ratio, the *P* value (obtained using the *z*-test defined in the SurvRM2 package in R, two-sided and not corrected for multiple comparisons) for the association and the additional time that affected people lived diagnosis-free are reported above each figure. The exact *P* values are 2.9 × 10^−15^ (**b**), 4.3 × 10^−41^ (**d**) and 2.3 × 10^−15^ (**e**). Shaded areas in **b**–**f** represent 95% CIs of the cumulative incidences (**b**,**d**–**f**) or the time-varying HR (**c**).

Here we used electronic health records (EHRs) and leveraged a natural experiment opportunity in the United States, created by the rapid uptake of the recombinant vaccine and the concurrent disuse of the live vaccine after October 2017 (Fig. 1a). By comparing the incidence of dementia in those who received a shingles vaccine just after versus just before this step change, we could accurately estimate the association between exposure to the recombinant vaccine and subsequent incidence of dementia diagnosis. We used propensity-score matching to further control for drifts in the characteristics of the vaccinated population.

A total of 103,837 individuals who received their first dose of shingles vaccine between November 2017 and October 2020 (95% received the recombinant vaccine; median (interquartile range, IQR) follow-up, 4.15 (3.16–4.99) years) were propensity-score matched to 103,837 individuals who received their first dose between October 2014 and September 2017 (98% received the live vaccine; median (IQR) follow-up, 6.0 (5.2–6.0) years; see Supplementary Table 1 for baseline characteristics, Supplementary Table 2 for person-year of follow-up and number of dementia cases and Supplementary Table 3 for the distribution of vaccinations per year). Individuals in the group that predominantly received the recombinant vaccine were at a lower risk of developing dementia over the next 6 years (restricted mean time lost (RMTL) ratio, 0.83; 95% confidence interval (CI), 0.80–0.87; *P* < 0.0001) than were those in the group that predominantly received the live vaccine, translating into 17% more time lived diagnosis-free, or 164 (95% CI 124–202) additional diagnosis-free days among those affected (Fig. 1b and Table 1).

**Table 1:** Summary of results for all analyses

	*n*	RMTL ratio (95% CI)	*P* value	Additional time lived diagnosis-free among affected people, days (95% CI)
**Propensity-score matched cohort studies**				
Primary analysis	103,837	0.83 (0.79–0.87)	2.9 × 10^−15^	164 (124–205)
Aligned follow-up horizons (cohort-wise)	103,837	0.83 (0.79–0.87)	4.3 × 10^−15^	165 (121–209)
Predominant vaccine	100,532	0.82 (0.79–0.86)	7.5 × 10^−16^	173 (131–214)
Adjusted for social deprivation	110,062	0.84 (0.80–0.88)	1.4 × 10^−14^	157 (117–196)
Excluding those who received both vaccines	66,998	0.79 (0.74–0.83)	1.5 × 10^−17^	214 (165–263)
Restricted exposure window (6 months)	20,243	0.83 (0.76–0.92)	0.00025	160 (74–246)
Females	54,846	0.78 (0.73–0.83)	2.3 × 10^−15^	222 (168–276)
Males	43,990	0.87 (0.81–0.94)	0.00028	122 (56–187)
**Other outcomes**				
Mortality	103,837	0.98 (0.95–1.01)	0.22	18 (−11–47)
Composite endpoint of dementia or mortality	103,837	0.93 (0.91–0.96)	3.8 × 10^−7^	64 (39–89)
Negative control outcome	103,837	0.97 (0.91–1.03)	0.29	32 (−27–90)
Herpes zoster infection	103,837	0.65 (0.61–0.69)	4.3 × 10^−41^	381 (326–435)
Herpes zoster infection (females)	54,846	0.64 (0.59–0.69)	1.4 × 10^−26^	393 (322–463)
Herpes zoster infection (males)	43,990	0.65 (0.58–0.72)	4.8 × 10^−15^	387 (293–482)
**Coarsened exact matched cohort studies**				
Parametric estimates of variance	82,102	0.82 (0.77–0.87)	1.6 × 10^−11^	192 (137–248)
Bootstrap estimates of variance	82,102	0.82 (0.79–0.86)	<0.001	192 (151–235)
Aligned follow-up horizons (pairwise)	82,102	0.85 (0.81–0.89)	<0.001	157 (111–203)

*n*, number of individuals in each cohort. The sample size for the analysis adjusted for social deprivation was slightly higher because it was conducted at a later point and TriNetX is a live network that continuously accrues data. The *P* values correspond to the *z*-test defined in the SurvRM2 package in R, except for the last two rows, for which a bootstrap test with 1,000 repetitions was used. All *P* values are two-sided and were not corrected for multiple comparisons.

**Extended Data Fig. 1:**
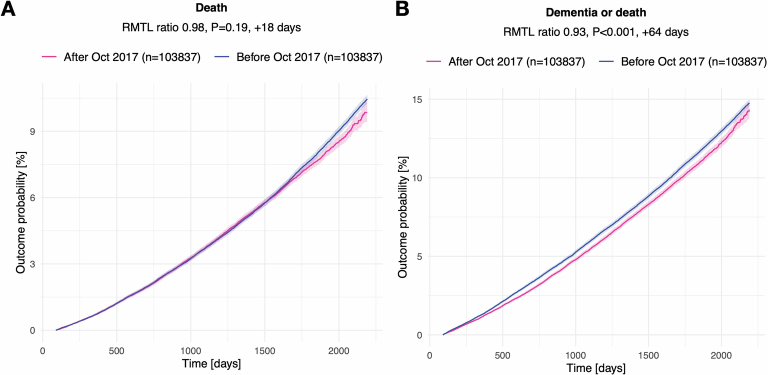
Kaplan-Meier curves for secondary outcomes in the 3 months-6 years after shingles vaccination in the primary analysis. The curves represent the Kaplan-Meier estimates of the cumulative incidence of death (**a**) and the composite outcome of death or dementia (**b**). Shaded areas around curves represent 95% confidence intervals. n = 103,837 in each cohort. P-values were obtained using the z-test defined in the SurvRM2 package in R, two-sided and not corrected for multiple comparisons. The exact p-values for (B) is 3.8×10^−7^.

**Extended Data Fig. 2:**
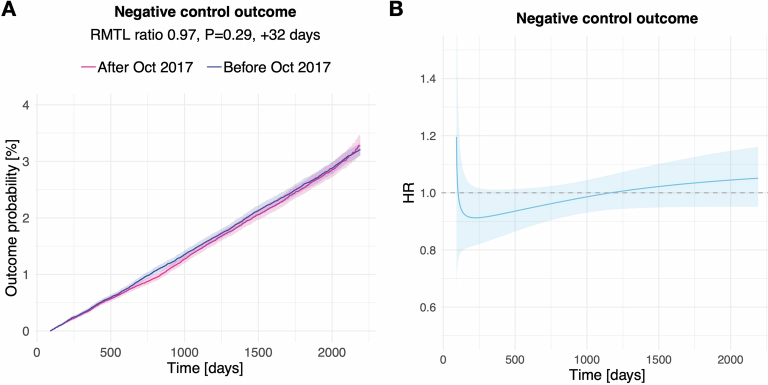
Results for the negative control outcome. (**a**) Curves representing the Kaplan-Meier estimates of the cumulative incidence of the negative control outcome in the 3 months-6 years after shingles vaccination. n = 103,837 in each cohort. The p-value was obtained using the z-test defined in the SurvRM2 package in R, two-sided and not corrected for multiple comparisons. (**b**) Curve representing the time-varying hazard ratio for the negative control outcome (n = 103,837 in each cohort). Shaded areas in A and B represent 95% confidence intervals.

The association was found consistently across dementia subcategories, except for frontotemporal and Lewy body dementia (Supplementary Table 4). Those vaccinated after October 2017 were also less likely to have a herpes zoster infection in the 6 years post-vaccination (RMTL ratio, 0.65; 95% CI, 0.61–0.69; *P* < 0.0001). There was no difference in negative control outcomes nor all-cause mortality, and results remained significant for the composite of dementia or death (Table 1 and Extended Data Figs. 1 and 2).

**Extended Data Fig. 3:**
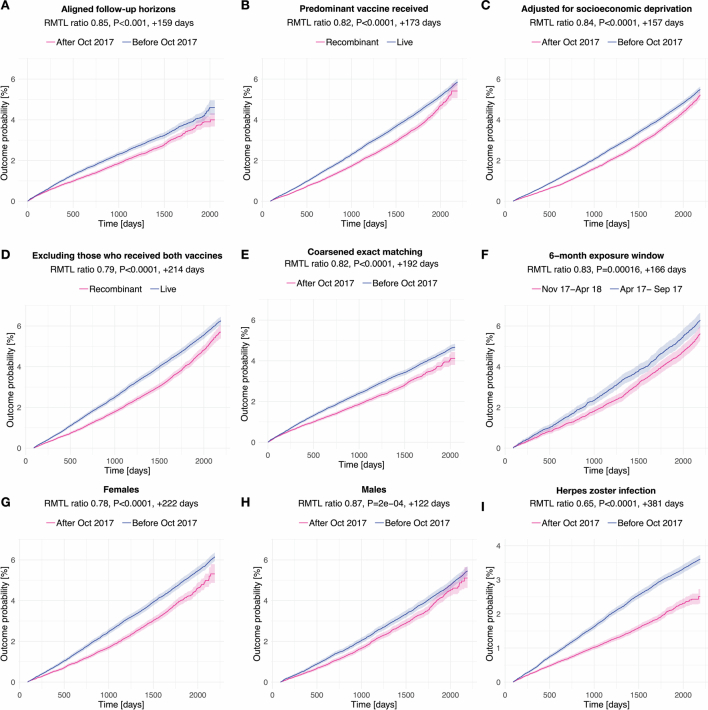
Kaplan-Meier curves showing the cumulative incidence of outcomes in the 3 months-6 years after shingles vaccination. (**a-h**) Results for dementia in the different robustness analyses. In (A), the results correspond to the coarsened exact matching with pairwise alignment of follow-up horizons. (**i**) Results for the incidence of herpes zoster infection. The ratio of restricted mean time lost (RMTL), the p-value for the association, and the additional time lived diagnosis-free among affected people are reported above each figure. Curves in all panels represent the Kaplan-Meier estimates of the cumulative incidence of the corresponding outcome. Shaded areas in all panels represent 95% confidence intervals. See Supplementary Tables 5–9 for baseline characteristics. The number of individuals in each cohort was respectively (A) 82102, (B) 100532, (C) 110062, (D) 66998, (E) 82102, (F) 20243, (G) 54846, (H) 43990, (I) 103837. P-values were obtained using the z-test defined in the SurvRM2 package in R (except for (A) where it was obtained using bootstrap with 1000 repetitions and is reported as <0.001 because all bootstrap replicates of the ratio of RMTL were below 1), two-sided and not corrected for multiple comparisons. The exact p-values are (B) 7.5×10^−16^, (C) 1.4×10^−14^, (D) 1.5×10^−17^, (E) 1.6×10^−11^, (G) 2.3×10^−15^, and (I) 4.3×10^−41^.

Similar results were found when cohorts were restricted to those who received the predominant vaccine; when exposure windows were limited to 6 months on either side of the step change; when individuals were excluded who received both vaccines; and when adjusting for socioeconomic deprivation (Table 1 and Extended Data Fig. 3). Comparable differences in risk were observed when the follow-up period was entirely before the COVID-19 pandemic (hazard ratio (HR), 0.74; 95% CI, 0.62–0.90; log-rank *P* = 0.0019; no evidence of violation of the proportional hazard assumption, *P* = 0.56). The results were also replicated when using coarsened exact matching for a core set of covariates (age, sex, race and neurological comorbidities), with consistent findings using parametric or bootstrap estimates of the variance (Table 1 and Extended Data Fig. 3). Aligning the follow-up horizons at the cohort level (in the primary analysis) and at the level of matched pairs of individuals (in the coarsened exact matching analysis) did not affect the results (Table 1; see Supplementary Tables 5–9 for baseline characteristics of secondary analyses).

The association between the recombinant shingles vaccine and dementia was found among both women and men (Fig. 1), and the effect was greater in women than in men (22% versus 13% more time lived diagnosis-free; permutation test: *P* = 0.017). The association with herpes zoster infection was also found in both women and men, but without moderation by sex (36% versus 35% more time lived diagnosis-free; permutation test: *P* = 0.87; Table 1).

**Extended Data Fig. 4:**
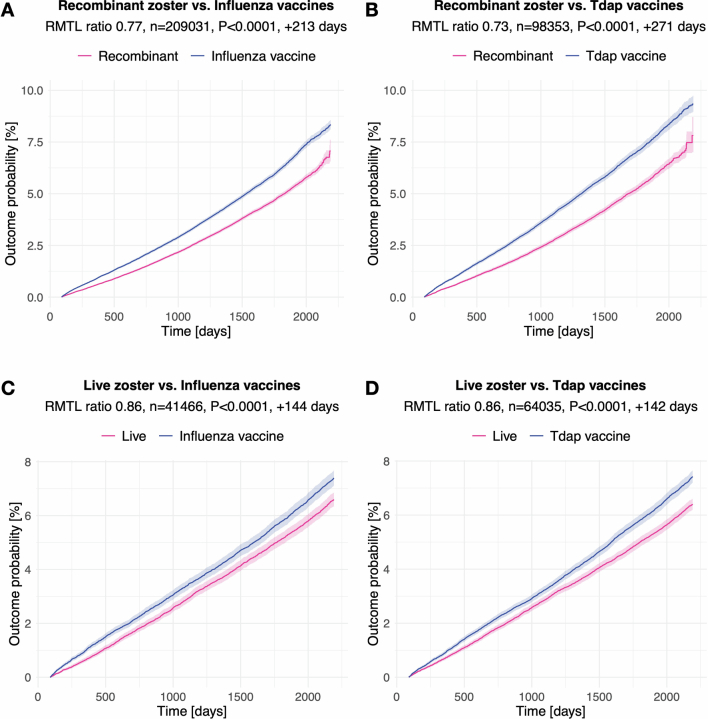
Kaplan-Meier curves for the comparisons between shingles vaccines and two other vaccines: influenza and Tdap. (**a-b**) Comparison with the recombinant shingles vaccine. (**c-d**) Comparison with live shingles vaccine. The ratio of restricted mean time lost (RMTL), the number of patients in each cohort, the bootstrap p-value for the association, and the additional time lived diagnosis-free among people affected are reported above each figure. Curves in all panels represent the Kaplan-Meier estimates of the cumulative incidence of the corresponding outcome. Shaded areas in all panels represent 95% confidence intervals. Baseline characteristics for these comparisons are provided in Supplementary Tables 11–14. The number of individuals in each cohort was respectively (A) 209031, (B) 98353, (C) 41466, and (D) 64035. P-values were obtained using the z-test defined in the SurvRM2 package in R, two-sided and not corrected for multiple comparisons. The exact p-values are (A) 1.4×10^−67^, (B) 2.6×10^−53^, (C) 1.2×10^−6^, and (D) 2.1×10^−9^.

Both shingles vaccines were associated with a lower risk of dementia than were the influenza and tetanus–diphtheria–pertussis (Tdap) vaccines (RMTL ratios, 0.73–0.86; all *P* < 0.0001; Extended Data Fig. 4 and Supplementary Table 10).

**Extended Data Fig. 5:**
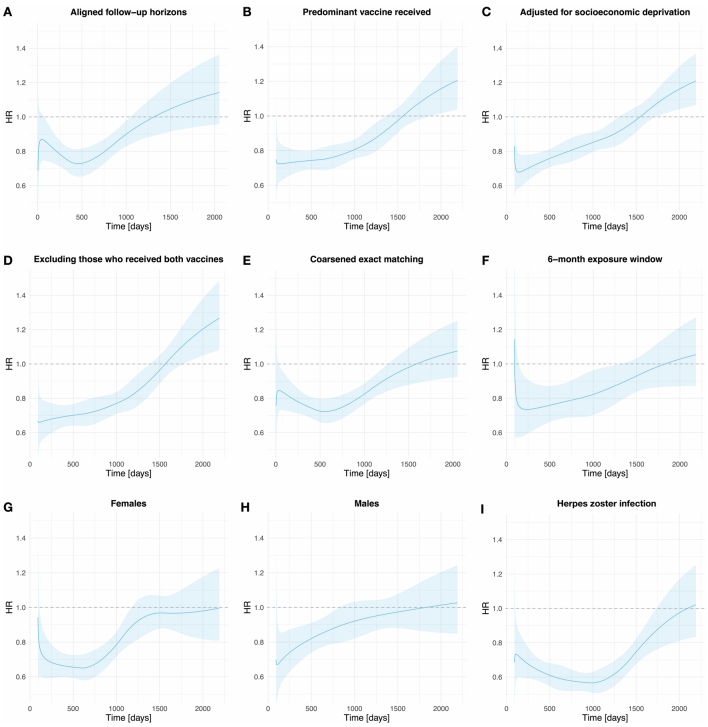
Time-varying hazard ratios (HR). Each curve represents the value of the HR from 3 months to 6 years post-vaccination. In (**A**), the results correspond to the coarsened exact matching with pairwise alignment of follow-up horizons. A HR < 1 indicates the risk is lower in those vaccinated predominantly with the recombinant vaccine. The shaded areas around the curves represent 95% CI. The number of individuals in each cohort was respectively (**A**) 82102, (**B**) 100532, (**C**) 110062, (**D**) 66998, (**E**) 82102, (**F**) 20243, (**G**) 54846, (**H**) 43990, (**I**) 103837.

The time-varying HRs became significantly lower than 1 within the first year of follow-up and then progressively approached (and, in some but not all robustness analyses, exceeded) 1 towards the end the 6-year follow-up (Fig. 1 and Extended Data Fig. 5), with differences in the shape of the curve apparent between men and women (Extended Data Fig. 5). The time-varying HR for the risk of herpes zoster infection followed a similar pattern (Extended Data Fig. 5).

## Discussion

Compared with the live vaccine, receiving the recombinant shingles vaccine is associated with a lower risk of developing dementia within the next 6 years. An increase by 17% in time lived without a dementia diagnosis (or 164 additional days among those later affected) is clinically meaningful and a particularly large effect size given that the live shingles vaccine is itself associated with a lower risk of dementia^3^, as replicated here. The consistency of the association in both sexes is important from a public-health perspective and for the credibility of the findings. No association between the live shingles vaccine and dementia was found in males in the natural experiment in Wales^3^, which called its causal interpretation into question^7^. In the present study, the protective effect was 9% greater in women than in men, which cannot be explained by better protection against shingles in women than men—a finding that merits further investigation.

This study is observational, and causality cannot be demonstrated. However, the rapid transition from the live to recombinant vaccine offered a window of opportunity to estimate associations with dementia without the main sources of selection bias^8^. The observation that all-cause mortality was very similar between cohorts, the lack of association with a composite negative control outcome and the robustness of the findings across several secondary analyses support the absence of obvious residual confounding. These results provide a rationale for conducting a randomized control trial aiming to confirm the findings and inform future cost–effectiveness analysis of the recombinant vaccine^1^.

The mechanisms by which the shingles vaccine might protect against dementia remain unclear. One explanation is that it protects against herpes infection, which itself causes dementia^9,10^. A link between herpes infections and dementia has been hypothesized for decades^11,12^. Although this hypothesis remains debated^13^, it would explain why both shingles vaccines are associated with lower risks of dementia, why the recombinant vaccine offers greater protection (it better protects against shingles^1^, as replicated in this study) and why the protective effect against dementia seems to wane towards later years of follow-up (as does the protective effect against herpes zoster infections). Additionally, the recombinant vaccine contains immunostimulants^14^ that could contribute to the protective effect against dementia. The observation that the time-varying HR became greater than 1 towards the end of the follow-up could imply that the vaccine delays, rather than prevents, dementia onset. However, this was not robustly observed across analyses (Extended Data Fig. 5) and therefore requires replication.

This study has several limitations in addition to those inherent to studies based on EHR data (such as no validation of diagnoses and sparse information on socioeconomic and lifestyle factors; see Supplementary Note 1). First, being diagnosis-free does not imply being disease-free, because there can be delays in diagnosis. However, if it is assumed that diagnostic delays are similar between cohorts, then differences in disease-free time will follow differences in diagnosis-free time. Second, we did not investigate the impact of multiple vaccine doses. Third, the number of people who received a shingles vaccine increased between before and after the introduction of the recombinant vaccine, justifying the need for additional control of covariates (as achieved here using matching). However, the fact that the association was maintained when the exposure window was reduced to 6 months on either side of the step change in recombinant-vaccine uptake argues strongly against the possibility that drifts in population characteristics could explain the main findings. Fourth, the paired nature of the data was not accounted for in the estimation of CIs within the primary analysis, an approach which is recommended by some authors^15^ but not others^16^. In any case, when this was accounted for in the secondary analysis using coarsened exact matching, little difference was observed in the estimated confidence intervals.

## Methods

### Study design and data source

We used EHR data from the TriNetX US Collaborative Network covering 62 healthcare organizations (hospitals, primary care and specialist providers) and including data from >100 million patients (Supplementary Note 1)^17^. Available data include demographics, diagnoses and medications. Data de-identification formally meets standards set by the Health Insurance Portability and Accountability Act Privacy Rule §164.514(b)(1). This study follows Strengthening the Reporting of Observational Studies in Epidemiology (STROBE) guidelines.

TriNetX is a platform that de-identifies and aggregates EHR data from contributing healthcare organizations (HCOs). No recruitment takes place. All patients who are seen at these HCOs have their data de-identified and incorporated into TriNetX. A typical organization will have a complex enterprise architecture in which the data flow through several databases, such as a data warehouse and a research data repository, on its way to TriNetX. TriNetX is a live platform, and data are continuously and regularly refreshed as soon as the HCOs themselves refresh their own data. HCOs update their data at various times, with more than 80% refreshing in 1-, 2- or 4-week frequency intervals. The average lag time for an HCO’s source data refresh is 1 month. TriNetX has been used in many prior studies, including a few that investigated dementia as an outcome^17–19^.

### Cohorts and exposures

Cohorts included all patients who received their first shingles vaccine dose at the age of 65 or older between 1 November 2017 and 31 October 2020 (primary cohort) and between 1 October 2014 and 30 September 2017 (comparator cohort). Patients were excluded if, before or up to one month after vaccination, they had any of the following diagnoses recorded in their health records:

- Vascular dementia (10th revision of the International Statistical Classification of Diseases and Related Health Problems (ICD-10) code F01)
- Dementia in other diseases classified elsewhere (F02)
- Unspecified dementia (F03)
- Parkinson’s disease (G20)
- Other degenerative diseases of the nervous system (G30–G32), which include all other dementias not mentioned above (for example Alzheimer’s disease (G30)).

Exclusion of those with a neurodegenerative disorder that was diagnosed within the first month since vaccination limits the impact of reverse causation owing to pre-existing (but undiagnosed) illness. Individuals vaccinated in October 2017 were excluded because this month marked the transition from the live vaccine to the recombinant vaccine.

### Covariates

Cohorts were matched for 60 covariates, including sociodemographic factors, comorbidities (capturing major body systems, and those associated with dementia), history of herpes infection and history of influenza vaccination. All covariates (with ICD-10 codes for comorbidities) are listed in Supplementary Table 1. Covariates were selected as follows.

All available sociodemographic factors were selected. These include age, sex (as recorded in the individual’s EHR), ethnicity, race and marital status. Age is reported as mean and s.d., but was matched using 2-year bins (65–66, 67–68, …) up to age 95; those 95 and older were grouped together. This provides tighter control on age than using it as a continuous variable.

All broad ICD-10 categories of comorbidities were then included to balance comorbidity profiles between cohorts and because an indirect link with dementia can be posited for most comorbidity profiles (for example, respiratory illness increases risk of infection and delirium and thus dementia; diseases of the ear can increase the risk of hearing loss which is a risk factor for dementia).

Some broad ICD-10 categories were further broken down into their most prevalent constituents. This includes ‘Neoplasms’ (ICD-10 codes C00–D49), which was deemed too heterogeneous (because it includes both benign and malignant neoplasms); cardiovascular diseases (I00–I99) and psychiatric disorders (F10–F59), given their strong link with dementia; and endocrine, nutritional and metabolic disorders (E00–E89), because they were deemed too heterogeneous and because they contain specific risk factors for dementia, such as overweight and obesity, diabetes, thyroid disorders and vitamin B deficiency. In addition, prior herpes infections (both herpes simplex and herpes zoster) were included as covariates.

Some factors affecting health and healthcare use (ICD-10 codes Z00–Z99) were also included on the basis of whether they differed substantially between unmatched cohorts (standardized mean difference (SMD) > 0.15) with a prevalence of at least 1 in 30 cases in either cohort.

Finally, to capture proxies of vaccine hesitancy, history of influenza vaccination (recommended every year for all adults in the United States) was included^20^.

### Outcomes

The primary outcome was a first diagnosis of dementia from 3 months (to exclude delayed diagnosis of pre-existing dementia) to 6 years post-vaccination in a time-to-event analysis. This included any of six ICD-10 codes: vascular dementia (ICD-10 code F01), dementia in other diseases classified elsewhere (F02), unspecified dementia (F03), Alzheimer’s disease (G30), frontotemporal dementia (G31.0), and dementia with Lewy bodies (G31.83), as in our previous studies^17^. Secondary outcomes included all-cause mortality (to assess whether vaccines were associated with overall differences in health), the composite of dementia or death (to assess for survivorship bias), each dementia subcategory and herpes zoster infections (ICD-10 code B02), as well as a composite negative control outcome of any acutely painful condition that was not associated with dementia (see Supplementary Note 2 for details).

### Statistical analyses

Propensity-score matching at a 1:1 ratio with a caliper of 0.1 was used to match cohorts on covariates. Characteristics with a standardized mean difference between cohorts of <0.1 were considered well matched^21^. The propensity score was calculated using a logistic regression (implemented by the function LogisticRegression of the scikit-learn package in Python 3.7) including each of the covariates mentioned above. To eliminate the influence of ordering of records, the record order in the covariate matrix was randomized before matching. The matching itself was performed using numpy 1.21.5 in Python 3.7.

Because most individuals vaccinated before October 2017 were matched to individuals vaccinated after October 2017 (but not vice versa), the estimand of the primary analysis is best interpreted as the average treatment effect in the controls.

Incidences of outcomes were calculated using the Kaplan–Meier estimator. The assumption that the hazards were proportional was tested using the generalized Schoenfeld approach implemented in the cox.zph function of the survival package (version 3.2.3) in R. In doing so, the proportionality assumption was found to be violated in the primary analysis (*P* < 0.0001). Consequently, the Cox proportional-hazard model was not used, and RMTL, which was calculated using R package survRM2 version 1.0.4, was used instead^22–24^.

The RMTL is the counterpart of restricted mean survival time^25,26^. The RMTL ratio has a meaningful clinical interpretation: it represents how much more time, on average, an individual has lived without the outcome during the follow-up period^22^. Unless otherwise stated, CIs were estimated using a parametric approach as defined in the SurvRM2 package in R^27^. Absolute differences in RMTL were translated into additional days lived without a diagnosis of dementia among those subsequently affected, calculated as the difference in RMTL divided by the cumulative incidence in the comparator cohort.

In addition, time-varying HRs were estimated using natural cubic splines fitted to the log-cumulative hazard^28^. This was achieved using the generalized survival models of the rstpm2 package (version 1.5.1) in R^29^. Splines with 1, 2 and 3 degrees of freedom were estimated for both the baseline log-cumulative hazard and its cohort dependency, and the number of degrees of freedom leading to the lowest Akaike Information Criterion (AIC) was selected.

Moderation by sex was tested using a permutation test, with 1,000 permutations as follows. The RMTL ratio between those vaccinated after versus before October 2017 were first calculated independently for men and women, and their difference was recorded. In each permutation, individuals were then randomly allocated to two groups of the same size as the initial ‘women’ and ‘men’ groups, and the analysis was repeated within these groups, thus leading to the calculation of RMTL ratios. The difference in absolute value between these RMTL ratios was recorded for each permutation, generating a distribution of 1,000 differences in RMTL ratios under the null hypothesis. The *P* value for the permutation test was calculated as:

$$P=\frac{1+{n}_{ > }}{1+n},$$

where *n* = 1,000 is the number of permutations, and *n*_>_ is the number of permutations for which the difference in RMTL ratios was greater (in absolute value) than that observed in the non-permuted dataset.

Because we used EHRs with coded health events, if an event was not present, it was considered absent. Missing data for sex, race and ethnicity were assigned their own category, which was included in the propensity-score matching, so that the matched cohorts had approximately equal numbers of patients with unknown sex, race and ethnicity.

Significance for all tests was set at two-sided *P* < 0.05. Statistical analyses were conducted in R 4.2.1.

### Secondary analysis

Analyses were repeated after: (1) stratification by sex, given the report that protective effects of the live vaccine were limited to women^3^; (2) restricting cohorts to those known to have received the predominant vaccine during each exposure window; (3) limiting exposure windows to 6 months before and after October 2017, to further decrease influences of drifts in population characteristics; (4) restricting, within the latter cohorts, the follow-up to 18 months so that it occurred entirely before the COVID-19 pandemic and is not subject to any effect that the pandemic might have had on diagnostic trends; (5) excluding those who received both vaccines; and (6) adjusting for socioeconomic deprivation (ICD-10 code Z59 ‘Problems related to housing and economic circumstances’).

Using a restricted set of key covariates (age, sex, race and neurological comorbidities), we then repeated the analysis using coarsened exact matching (to control for non-linear effects and interactions in these confounding factors)^30^, and comparing both parametric and bootstrap (with 1,000 resampling of pairs of matched individuals) estimates of variance (to assess the effect of respecting the paired nature of the data on variance estimates)^16,31^.

In addition, to assess whether observed associations were an artifact of the differences in follow-up times between cohorts, analyses were repeated after aligning follow-up times (at the cohort level in the primary analysis, and at the level of pairs of individuals in the analysis based on coarsened exact matching).

Both shingles vaccines were also compared with Tdap and influenza vaccines to control for non-specific effects of vaccination. These vaccines were given in the same exposure windows as the primary cohorts (for example, when comparing the recombinant vaccine with the influenza vaccine, the cohort that took the influenza vaccine received it between 1 November 2017 and 31 October 2020). The estimands are best interpreted as conditional average treatment effects (conditional on being in the subpopulation for which covariates overlap between cohorts), because only subgroups within each cohort were successfully matched to each other.

Supplementary Note 3 contains further details on secondary analyses.

### Reporting summary

Further information on research design is available in the Nature Portfolio Reporting Summary linked to this article.

## Online content

Any methods, additional references, Nature Portfolio reporting summaries, source data, extended data, supplementary information, acknowledgements, peer review information; details of author contributions and competing interests; and statements of data and code availability are available at 10.1038/s41591-024-03201-5.

## Supplementary information

Supplementary Information Supplementary Notes 1–3 and Tables 1–14.

Reporting Summary

## Data Availability

The TriNetX system returned the results of these analyses as csv files, which we downloaded and archived. Aggregate data, as presented in this article, can be freely accessed at https://osf.io/9frxm/. The data used for this article were acquired from TriNetX. This study had no special privileges. Inclusion criteria specified in Methods would allow other researchers to identify similar cohorts of patients as we used here for these analyses; however, TriNetX is a live platform with new data being added daily, so exact counts will vary. To gain access to the data, a request can be made to TriNetX (join@trinetx.com), but costs might be incurred, and a data-sharing agreement will be necessary.

## Code availability

The code used to generate the results of this analysis can be freely accessed at https://osf.io/9frxm/.
