# Supplementary material for: The recombinant shingles vaccine is associated with lower risk of dementia
Source: Nat Med. 2024 Jul 25;30(10):2777–81. doi: 10.1038/s41591-024-03201-5 (PMC11485228; doi:10.1038/s41591-024-03201-5)
Supplement: Supplementary file 1 — Supplementary Notes 1–3 and Tables 1–14. [file 41591_2024_3201_MOESM1_ESM.pdf]

nature medicine

Supplementary information https://doi.org/10.1038/s41591-024-03201-5

The recombinant shingles vaccine is associated with lower risk of dementia

In the format provided by the authors and unedited

Supplementary Material

Supplementary Note 1: TriNetX US Collaborative Network

This section largely replicates our previous description of the network.[1]

Legal and ethical status
TriNetX's networks are compliant with the Health Insurance Portability and Accountability Act (HIPAA), the US federal law which protects the privacy and security of healthcare data. TriNetX is certified to the ISO 27001:2013 standard and maintains an Information Security Management System (ISMS) to ensure the protection of the healthcare data it has access to and to meet the requirements of the HIPAA Security Rule. Any data displayed on the TriNetX Platform in aggregate form, or any patient level data provided in a data set generated by the TriNetX Platform, only contains de-identified data as per the de-identification standard defined in Section §164.514(a) of the HIPAA Privacy Rule. The process by which the data is de-identified is attested to through a formal determination by a qualified expert as defined in Section §164.514(b)(1) of the HIPAA Privacy Rule. This formal determination by a qualified expert, refreshed in December 2020, supersedes the need for TriNetX's previous waiver from the Western Institutional Review Board (IRB). The network contains data that are provided by participating Health Care Organizations (HCOs), each of which represents and warrants that it has all necessary rights, consents, approvals and authority to provide the data to TriNetX under a Business Associate Agreement (BAA), so long as their name remains anonymous as a data source and their data are utilized for research purposes. The data shared through the TriNetX Platform are attenuated to ensure that they do not include sufficient information to facilitate the determination of which HCO contributed which specific information about a patient. Keeping the identity of participating HCOs from the researchers using the data also contribute to complying with legal frameworks and ethical guidelines guarding against data re-identification.

Acquisition of data, quality control, and other procedures
The data are stored onboard a TriNetX appliance – a physical server residing at the institution's data centre or a virtual hosted appliance. The TriNetX platform is a fleet of these appliances connected into a federated network able to broadcast queries to each appliance. Results are subsequently collected and aggregated.

Once the data are sent to the network, they are mapped to a standard and controlled set of clinical terminologies and undergo a data quality assessment including 'data cleaning' that rejects records which do not meet the TriNetX quality standards. HIPAA compliance of the clinical patient data is achieved using de-identification. Different data modalities are available in the network. They include demographics (coded to HL7 version 3 administrative standards), diagnoses (represented by ICD-10-CM codes), procedures (coded in ICD-10-PCS or CPT), and measurements (coded to LOINC).

The data from a typical HCO generally go back around 9 years, with some going back 15 years. The data are continuously updated. HCOs update their data at various times, with most refreshing every 1, 2, or 4 weeks.

Data quality assessment followed a standardised strategy wherein the data are reviewed for conformance (adherence to specified standards and formats), completeness (quantifying data presence or absence) and plausibility (believability of the data from a clinical perspective). There are pre-defined metrics for each of the above assessment categories. Results for these metrics are visualised and reviewed for each new site that joins the network as well as on an ongoing basis. Any identified issue is communicated to the data provider and resolved before continuing data collection.

The basic formatting of contributed data is also checked (e.g. to ensure that dates are properly represented). Records are checked against a list of required fields (e.g., patient identifier) and rejects those records for which the required information is missing. Referential integrity checking is done to

ensure that data spanning multiple database tables can be successfully joined together. As the data are refreshed, changes in volume of data over time is monitored to ensure data validity. At least one non-demographic fact for each patient is required for them to be counted in the dataset. Patient records with only demographics information are discarded.

The software also undergoes quality control. The engineers testing the software are independent from the engineers developing it. Each test code is checked by two independent testing engineers. Each piece of software is tested extensively against a range of synthetic data (i.e. generated for the purpose of testing) for which the expected output is established independently. If the software fails to return this output, then the software is deemed to have failed the test and is examined and modified accordingly. For statistical software (including that used for propensity score matching, for Kaplan-Meier analysis, etc), an additional quality control step is implemented. Two independent codes are written in two different programming languages (typically R and python) and the statistical results are compared. If discrepancies are identified, then the codes are deemed to have failed the test and are examined and modified accordingly. All the code is reviewed independently by another engineer.

The test strategy follows three levels of granularity:

1. Unit tests: These test specific blocks, or units, of code that perform specific actions (e.g. querying the database).
2. Integration tests: These ensure that different components are working together correctly.
3. End-to-end tests: These tests run the entire system and check the final output.

Mortality data
TriNetX records death from EHR which accurately record in-hospital deaths as well as some out-of-hospital deaths. For a subset of patients, linkage with third-party mortality data sources (SSA, Private Obituary Data, and Private Claims Data) is used to increase coverage of out-of-hospital deaths.

Some comments on advantages and disadvantages of EHR data
One advantage of EHR data, like those in TriNetX, over insurance claim data is that both insured and uninsured patients are included. An advantage of EHR data over survey data is that they represent the diagnostic rates in the population presenting to healthcare facilities. This provides an accurate account of the burden of specific diagnoses on healthcare systems. However, there are also limitations inherent to research of this kind using electronic health records,[2–4] including TriNetX:

1. Despite the matching and use of various comparison cohorts, there may well be residual confounding, particularly related to social and economic factors which are not well captured in EHR networks and which might influence outcomes post-vaccination as well as choice of zoster vaccine.
2. We do not know which diagnoses were made in primary or secondary care or specialist facilities, nor by whom.
3. A patient may be seen in different HCOs for different parts of their care, and if one HCO is not part of the federated network then part of their medical records may not be available. Using a network of HCOs (rather than a single HCO) limits this possibility but does not fully eliminate it.
4. Since the data are presented as they are recorded, we cannot be sure that there has not been mis-recording of information, adding a degree of noise to the data.
5. Historical data before the start of EHRs (or the addition of an HCO to the network) may well be incomplete.

Supplementary Note 2: Definition of negative control outcome

In studies with dementia as the primary outcome, identifying an appropriate negative control outcome is challenging because cognitive impairment tends to affect many aspects of health. An individual

with cognitive impairment might pay less attention to relatively benign health problems or might receive less medical attention for them, and therefore they might be less likely to have such health problems recorded in their EHR. Conversely, they might be receiving support for their personal care and this might lead to relatively benign conditions being more easily identified and diagnosed. As a result, many outcomes likely correlate positively or negatively with a diagnosis of dementia.

We reasoned that outcomes that are less likely to be affected by such covariation are those that are not typically associated with dementia and that almost invariably require medical attention. Several acutely painful conditions meet these criteria. The UK National Health Service had compiled a list of 20 painful health conditions (https://familyserviceshub.havering.gov.uk/kb5/havering/directory/advice.page?id=nsFR_oRkZbU):

- Acute pancreatitis
- Appendicitis
- Arthritis
- Broken bones
- Cluster headaches
- Complex regional pain syndrome (CRPS)
- Endometriosis
- Fibromyalgia
- Frozen shoulder
- Gout
- Myocardial infarction
- Renal stones
- Migraine
- Pain after surgery
- Sciatica
- Shingles
- Sickle cell disease
- Slipped disc
- Stomach ulcer
- Trigeminal neuralgia

From them, we excluded those that tend to be chronic or recurrent (e.g. migraine), those that only affect women or specific clinical scenarios (e.g., endometriosis, post-surgical pain), those that mostly affect younger people (e.g. cluster headache), and those associated with dementia (all those related to falls or musculoskeletal impairment, dehydration such as kidney stones, cardiovascular health such as myocardial infarction, peptic ulcers,[5] and gout[6]). This left us with a list of four conditions to which we added cholecystitis (as a replacement for renal stones that is associated with dehydration):

- Acute pancreatitis (ICD-10 code K81.0)
- Appendicitis (K35)
- Acute cholecystitis (K81.0)
- Adhesive capsulitis of the shoulder (M75.0)
- Trigeminal neuralgia (G50.0)

The negative control outcome was then defined as the composite outcome of a first diagnosis of any of these outcomes.

Supplementary Note 3: Details on statistical analyses

As a secondary analysis, we used coarsened exact matching (CEM), an alternative to propensity score matching with better model independence properties (i.e. it better controls for non-linear effects of the

covariates as well as interactions between covariates).[7] Because it is more affected by the curse of dimensionality (the more covariates are included, the fewer matches are found) than propensity-score matching, this analysis was limited to four core covariates: age (stratified in bins of 3 years: 65-67, 68-70, 71-73, 74-76 years-old), sex (male vs female), race (white vs non-white), and neurological comorbidities (presence vs absence of a G00-G99 code).

Within this analysis, we also tested the impact of estimating variance of the ratio of restricted mean time lost using bootstrap (in which pairs of matched individuals are resampled with replacement 1000 times) rather than the parametric approach as described in the main manuscript.

Those vaccinated after October 2017 had shorter follow-ups on average than those vaccinated before October 2017. We tested whether this impacted the results by aligning follow-up times. This was achieved by selecting pairs of individuals (one from each cohort) and censoring the one with the longest follow-up at the time of last follow-up of the other. In CEM, this was achieved at the level of pairs of matched individuals. For the primary analysis based on propensity score matching, the identity of which individual is matched with which is not released by TriNetX for patient's privacy reason, and only the pooled life table of matched subjects is available. Alignment of follow-up times was therefore achieved at the cohort level by selecting pairs of individuals at random, thereby guaranteeing that the distributions of follow-up times were identical between matched cohorts.

Supplementary Table 1 Baseline characteristics for the primary analysis showing both the distribution before and after matching. SMD=Standardised mean difference.

	Before matching			After matching		
	**After Oct 2017**	**Before Oct 2017**	**SMD**	**After Oct 2017**	**Before Oct 2017**	**SMD**
**Number**	386023	103958	-	103837	103837	-
**SOCIODEMOGRAPHICS**						
Age; mean (SD); y	71.97 (5.41)	70.70 (4.80)	0.25	70.65 (4.79)	70.70 (4.80)	0.012
Sex; n (%)						
Female	209636 (54.31)	55906 (53.78)	0.011	55474 (53.42)	55836 (53.77)	0.007
Male	157966 (40.92)	44013 (42.34)	0.029	44420 (42.78)	43962 (42.34)	0.0089
Other/Unknown	18421 (4.77)	4039 (3.88)	0.044	3943 (3.80)	4039 (3.89)	0.0048
Race; n (%)						
White	291437 (75.50)	73745 (70.94)	0.1	74256 (71.51)	73730 (71.01)	0.011
Black or African American	27804 (7.20)	11252 (10.82)	0.13	11443 (11.02)	11190 (10.78)	0.0078
Asian	19257 (4.99)	3943 (3.79)	0.058	3853 (3.71)	3943 (3.80)	0.0046
Other	8492 (2.20)	2570 (2.47)	0.018	2406 (2.32)	2564 (2.47)	0.01
Unknown	36847 (9.54)	11770 (11.32)	0.058	11140 (10.73)	11735 (11.30)	0.018
Ethnicity; n (%)						
Hispanic or Latino	13929 (3.61)	5249 (5.05)	0.071	5141 (4.95)	5220 (5.03)	0.0035
Not Hispanic of Latino	302893 (78.47)	78615 (75.62)	0.068	79981 (77.03)	78533 (75.63)	0.033
Unknown	69008 (17.88)	20053 (19.29)	0.036	18668 (17.98)	20043 (19.30)	0.034
Marital status; n (%)						
Married	111497 (28.88)	29823 (28.69)	0.0043	29839 (28.74)	29792 (28.69)	0.001
Never married	21039 (5.45)	6595 (6.34)	0.038	6585 (6.34)	6570 (6.33)	0.00059
Divorced	17053 (4.42)	5753 (5.53)	0.051	5715 (5.50)	5727 (5.51)	0.00051
Domestic partner	332 (0.086)	75 (0.072)	0.0049	72 (0.069)	75 (0.072)	0.0011
**COMORBIDITIES [ICD-10 code]; n (%)**						

Infections and parasites [A00-B99]	118487 (30.69)	24160 (23.24)	0.17	24450 (23.55)	24146 (23.25)	0.0069
Herpes simplex infection [B00]	9539 (2.47)	1401 (1.35)	0.082	1400 (1.35)	1401 (1.35)	8.3e-05
Herpes zoster infection [B02]	18150 (4.70)	4199 (4.04)	0.032	4272 (4.11)	4195 (4.04)	0.0037
Neoplasms (benign and malignant) [C00-D49]	172128 (44.59)	34960 (33.63)	0.23	35307 (34.00)	34949 (33.66)	0.0073
Skin cancer [C43-44]	30969 (8.02)	5164 (4.97)	0.12	5260 (5.07)	5164 (4.97)	0.0042
In situ neoplasms [D00-09]	15290 (3.96)	2516 (2.42)	0.088	2529 (2.44)	2516 (2.42)	0.00081
Benign neoplasms [D10-36]	118479 (30.69)	22751 (21.89)	0.2	22963 (22.11)	22747 (21.91)	0.005
Neoplasms of uncertain behaviour [D37-38]	43461 (11.26)	8291 (7.97)	0.11	8311 (8.00)	8288 (7.98)	0.00082
Haematological conditions [D50-89]	102646 (26.59)	20883 (20.09)	0.15	21100 (20.32)	20866 (20.09)	0.0056
Thyroid disorders [E00-07]	89080 (23.08)	18603 (17.89)	0.13	18762 (18.07)	18591 (17.90)	0.0043
Iodine-deficiency disorder [E01]	9413 (2.44)	2807 (2.70)	0.017	2845 (2.74)	2798 (2.69)	0.0028
Hypothyroidism [E03]	68969 (17.87)	14547 (13.99)	0.11	14610 (14.07)	14538 (14.00)	0.002
Nontoxic goiter [E04]	23376 (6.06)	4409 (4.24)	0.082	4526 (4.36)	4406 (4.24)	0.0057
Hyperthyroidism [E05]	7909 (2.05)	1733 (1.67)	0.028	1760 (1.70)	1733 (1.67)	0.002
Other disorders of thyroid [E07]	12180 (3.15)	2779 (2.67)	0.029	2800 (2.70)	2775 (2.67)	0.0015
Diabetes mellitus [E08-13]	88471 (22.92)	24982 (24.03)	0.026	25267 (24.33)	24903 (23.98)	0.0082
Vitamin B deficiency [E53]	20320 (5.26)	3751 (3.61)	0.08	3762 (3.62)	3750 (3.61)	0.00062
Vitamin D deficiency [E55]	83446 (21.62)	16418 (15.79)	0.15	16598 (15.98)	16415 (15.81)	0.0048
Overweight and obesity [E66]	84884 (21.99)	17087 (16.44)	0.14	17389 (16.75)	17081 (16.45)	0.008
Metabolic disorders [E70-88]	261649 (67.78)	61101 (58.77)	0.19	61815 (59.53)	61047 (58.79)	0.015
Psychiatric comorbidities						
Substance use disorder [F10-19]	40989 (10.62)	10792 (10.38)	0.0077	10981 (10.57)	10763 (10.37)	0.0069
Mood disorder [F30-39]	70708 (18.32)	16271 (15.65)	0.071	16471 (15.86)	16250 (15.65)	0.0058
Anxiety disorder [F40-48]	82357 (21.34)	16454 (15.83)	0.14	16637 (16.02)	16446 (15.84)	0.005
Organic disorder [F50-59]	20330 (5.27)	3104 (2.99)	0.11	3172 (3.06)	3104 (2.99)	0.0038
Neurological disorder [G00-99]	193383 (50.10)	41433 (39.86)	0.21	41860 (40.31)	41406 (39.88)	0.0089
Ophthalmic disorder [H00-59]	142443 (36.90)	30591 (29.43)	0.16	31281 (30.12)	30577 (29.45)	0.015
Ear/mastoid process disease [H60-95]	113529 (29.41)	22171 (21.33)	0.19	22281 (21.46)	22165 (21.35)	0.0027

Cardiovascular disease [I00-99]	271987 (70.46)	67809 (65.23)	0.11	68511 (65.98)	67703 (65.20)	0.016
Chronic rheumatic heart diseases [I05-09]	15330 (3.97)	3352 (3.22)	0.04	3486 (3.36)	3349 (3.23)	0.0074
Hypertension [I10]	229687 (59.50)	58437 (56.21)	0.067	59134 (56.95)	58336 (56.18)	0.016
Ischaemic heart disease [I20-25]	69556 (18.02)	16814 (16.17)	0.049	17059 (16.43)	16780 (16.16)	0.0073
Other heart disease [I30-52]	113576 (29.42)	24615 (23.68)	0.13	24840 (23.92)	24584 (23.68)	0.0058
Cerebrovascular diseases [I60-69]	37017 (9.59)	8927 (8.59)	0.035	9088 (8.75)	8903 (8.57)	0.0063
Cerebral infarction [I63]	12338 (3.20)	3299 (3.17)	0.0013	3362 (3.24)	3281 (3.16)	0.0044
Atherosclerosis [I70]	23305 (6.04)	4522 (4.35)	0.076	4691 (4.52)	4520 (4.35)	0.008
Other peripheral vascular diseases [I73]	23286 (6.03)	5388 (5.18)	0.037	5511 (5.31)	5379 (5.18)	0.0057
Other disorders of arteries/arterioles [I77]	16535 (4.28)	2686 (2.58)	0.093	2759 (2.66)	2685 (2.59)	0.0045
Disease of veins/lymphatics [I80-89]	44204 (11.45)	9082 (8.74)	0.09	9229 (8.89)	9076 (8.74)	0.0052
Unspecified disease of circulatory system [I95-99]	26056 (6.75)	5480 (5.27)	0.062	5649 (5.44)	5473 (5.27)	0.0075
Respiratory disease [J00-99]	197712 (51.22)	43986 (42.31)	0.18	44565 (42.92)	43954 (42.33)	0.012
Digestive disease [K00-95]	224875 (58.25)	49786 (47.89)	0.21	50111 (48.26)	49743 (47.91)	0.0071
Dermatological disease [L00-99]	176463 (45.71)	36675 (35.28)	0.21	37151 (35.78)	36656 (35.30)	0.01
Musculoskeletal disease [M00-99]	279190 (72.33)	64577 (62.12)	0.22	65258 (62.85)	64522 (62.14)	0.015
Genitourinary disease [N00-99]	211464 (54.78)	46788 (45.01)	0.2	47325 (45.58)	46745 (45.02)	0.011
Congenital malformation [Q00-99]	33732 (8.74)	7000 (6.73)	0.075	7115 (6.85)	6996 (6.74)	0.0046
Injury/poisoning [S00-T88]	157555 (40.81)	32042 (30.82)	0.21	32403 (31.21)	32026 (30.84)	0.0078
Falls [W00-19]	27774 (7.20)	4894 (4.71)	0.11	4965 (4.78)	4894 (4.71)	0.0032
**FACTORS AFFECTING HEALTH**						
History of coded normal general examination	168684 (43.70)	35403 (34.05)	0.2	35707 (34.39)	35378 (34.07)	0.0067
History of coded abnormal general examination	14434 (3.74)	659 (0.63)	0.21	676 (0.65)	659 (0.64)	0.002
History of cancer screening	163113 (42.26)	34590 (33.27)	0.19	34907 (33.62)	34561 (33.28)	0.0071
Long-term drug therapy	107902 (27.95)	17730 (17.05)	0.26	17931 (17.27)	17726 (17.07)	0.0052
History of nicotine dependence	54130 (14.02)	7569 (7.28)	0.22	7680 (7.40)	7569 (7.29)	0.0041
Prior influenza vaccine	212320 (55.00)	41826 (40.23)	0.3	42112 (40.56)	41825 (40.28)	0.0056

Supplementary Table 2 Follow-up time in person-years (p.y.), number of dementia cases, and number lost to follow-up (e.g. because they made no further contact with their healthcare provider or moved to a different area). These data are presented for each year of follow-up and for both the primary analysis and after alignment of follow-up horizons.

	Year 1	Year 2	Year 3	Year 4	Year 5	Year 6
**Primary analysis**						
Before Nov 17	101849 p.y.	97872 p.y.	93639 p.y.	88581 p.y.	82731 p.y.	75421 p.y.
	636 cases	932 cases	940 cases	954 cases	947 cases	988 cases
	3304 lost	3072 lost	3696 lost	4488 lost	5371 lost	7860 lost
After Nov 17	100576 p.y.	94437 p.y.	86882 p.y.	69162 p.y.	40394 p.y.	11815 p.y.
	459 cases	625 cases	752 cases	671 cases	470 cases	180 cases
	5956 lost	5463 lost	9321 lost	23867 lost	30524 lost	24557 lost
**Aligned follow-up**						
Before Nov 17	98666 p.y.	89016 p.y.	78352 p.y.	59041 p.y.	32297 p.y.	8758.4 p.y.
	616 cases	844 cases	783 cases	636 cases	375 cases	111 cases
	9513 lost	8509 lost	12147 lost	23964 lost	26827 lost	18863 lost
After Nov 17	98666 p.y.	89016 p.y.	78352 p.y.	59041 p.y.	32297 p.y.	8758.4 p.y.
	452 cases	584 cases	688 cases	583 cases	371 cases	135 cases
	9677 lost	8769 lost	12242 lost	24017 lost	26831 lost	18839 lost

Supplementary Table 3 Number of people receiving either vaccination per year in both cohorts (years before October 2017 represent the control cohort and years after October 2017 represent the main cohort of interest) of the primary analysis. Note that numbers differ slightly from the primary analysis as this table was generated at a later point and TriNetX is a live network with continuously accruing data.

Year	Number of people receiving either vaccine
**Oct 2014-Sep 2015**	40568
**Oct 2015-Sep 2016**	36484
**Oct 2016-Sep 2017**	33175
**Nov 2017-Oct 2018**	143862
**Nov 2018-Oct 2019**	155711
**Nov 2019-Oct 2020**	113168

Supplementary Table 4 Results for dementia subcategories.

Outcome	RMTL ratio (95% CI)	p-value	Additional time lived diagnosis-free among affected people, days (95% CI)
Alzheimer's	0.84 (0.78-0.90)	6.4e-06	152 (87-218)
Vascular dementia	0.79 (0.71-0.87)	2.1e-06	186 (110-262)
Dementia in diseases classified elsewhere	0.90 (0.84-0.96)	0.0024	93 (33-152)
Unspecified dementia	0.80 (0.76-0.85)	1.7e-15	186 (141-232)
Frontotemporal dementia	1.22 (0.93-1.61)	0.15	-229 (-543 - 85)
Lewy body dementia	0.91 (0.71-1.16)	0.44	71 (-109 - 252)

RMTL=Restricted mean time lost. The p-values correspond to the z-test defined in the SurvRM2 package in R, two-sided and not corrected for multiple comparisons.

Supplementary Table 5 Baseline characteristics for the analysis based on predominant vaccine received. SMD=Standardised mean difference.

	Recombinant	Live	SMD
**Number**	100532	100532	-
**SOCIODEMOGRAPHICS**			
Age; mean (SD); y	70.6 (4.8)	70.7 (4.8)	0.013
Sex; n (%)			
Female	53101 (52.8)	53571 (53.3)	0.0094
Male	43322 (43.1)	42919 (42.7)	0.0081
Other/Unknown	4109 (4.1)	4042 (4.0)	0.0034
Race; n (%)			
White	72353 (72.0)	71894 (71.5)	0.01
Black or African American	10766 (10.7)	10643 (10.6)	0.004
Asian	3742 (3.7)	3794 (3.8)	0.0027
Other	1849 (1.8)	2010 (2.0)	0.012
Unknown	11137 (11.1)	11572 (11.5)	0.014
Ethnicity; n (%)			
Hispanic or Latino	4787 (4.8)	4886 (4.9)	0.0046
Not Hispanic of Latino	76669 (76.3)	75290 (74.9)	0.032
Unknown	19076 (19.0)	20356 (20.2)	0.032
Marital status; n (%)			
Married	30938 (30.8)	31183 (31.0)	0.0053
Never married	6684 (6.6)	6683 (6.6)	4e-05
Divorced	5762 (5.7)	5785 (5.8)	0.00098
Domestic partner	82 (0.082)	74 (0.074)	0.0029
**COMORBIDITIES [ICD-10 code]; n (%)**			
Infections and parasites [A00-B99]	23233 (23.1)	22922 (22.8)	0.0074
Herpes simplex infection [B00]	1262 (1.3)	1300 (1.3)	0.0034
Herpes zoster infection [B02]	4105 (4.1)	4038 (4.0)	0.0034
Neoplasms (benign and malignant) [C00-D49]	33280 (33.1)	33243 (33.1)	0.00078
Skin cancer [C43-44]	4887 (4.9)	4962 (4.9)	0.0035
In situ neoplasms [D00-09]	2352 (2.3)	2411 (2.4)	0.0039
Benign neoplasms [D10-36]	21282 (21.2)	21285 (21.2)	7.3e-05
Neoplasms of uncertain behaviour [D37-38]	7528 (7.5)	7593 (7.6)	0.0025
Haematological conditions [D50-89]	20084 (20.0)	19937 (19.8)	0.0037
Thyroid disorders [E00-07]	17824 (17.7)	17801 (17.7)	6e-04
Iodine-deficiency disorder [E01]	2609 (2.6)	2657 (2.6)	0.003
Hypothyroidism [E03]	14052 (14.0)	14052 (14.0)	0
Nontoxic goiter [E04]	4087 (4.1)	4083 (4.1)	2e-04
Hyperthyroidism [E05]	1744 (1.7)	1699 (1.7)	0.0035
Other disorders of thyroid [E07]	2691 (2.7)	2578 (2.6)	0.007
Diabetes mellitus [E08-13]	24507 (24.4)	24112 (24.0)	0.0092
Vitamin B deficiency [E53]	3481 (3.5)	3468 (3.5)	0.00071

Vitamin D deficiency [E55]	15354 (15.3)	15336 (15.3)	5e-04
Overweight and obesity [E66]	16532 (16.4)	16371 (16.3)	0.0043
Metabolic disorders [E70-88]	59044 (58.7)	58299 (58.0)	0.015
Psychiatric comorbidities			
Substance use disorder [F10-19]	10729 (10.7)	10441 (10.4)	0.0093
Mood disorder [F30-39]	16021 (15.9)	15680 (15.6)	0.0093
Anxiety disorder [F40-48]	15782 (15.7)	15802 (15.7)	0.00055
Organic disorder [F50-59]	3019 (3.0)	2924 (2.9)	0.0056
Neurological disorder [G00-99]	39831 (39.6)	39636 (39.4)	0.004
Ophthalmic disorder [H00-59]	29383 (29.2)	29067 (28.9)	0.0069
Ear/mastoid process disease [H60-95]	21097 (21.0)	20954 (20.8)	0.0035
Cardiovascular disease [I00-99]	65942 (65.6)	65086 (64.7)	0.018
Chronic rheumatic heart diseases [I05-09]	3045 (3.0)	2881 (2.9)	0.0096
Hypertension [I10]	57007 (56.7)	56307 (56.0)	0.014
Ischaemic heart disease [I20-25]	16426 (16.3)	16103 (16.0)	0.0087
Other heart disease [I30-52]	23987 (23.9)	23592 (23.5)	0.0092
Cerebrovascular diseases [I60-69]	8717 (8.7)	8613 (8.6)	0.0037
Cerebral infarction [I63]	3224 (3.2)	3213 (3.2)	0.00062
Atherosclerosis [I70]	4655 (4.6)	4509 (4.5)	0.007
Other peripheral vascular diseases [I73]	5244 (5.2)	5153 (5.1)	0.0041
Other disorders of arteries/arterioles [I77]	2571 (2.6)	2547 (2.5)	0.0015
Disease of veins/lymphatics [I80-89]	8745 (8.7)	8633 (8.6)	0.004
Unspecified disease of circulatory system [I95-99]	5405 (5.4)	5233 (5.2)	0.0076
Respiratory disease [J00-99]	42785 (42.6)	42317 (42.1)	0.0094
Digestive disease [K00-95]	48127 (47.9)	47724 (47.5)	0.008
Dermatological disease [L00-99]	35157 (35.0)	34740 (34.6)	0.0087
Musculoskeletal disease [M00-99]	62558 (62.2)	61830 (61.5)	0.015
Genitourinary disease [N00-99]	45321 (45.1)	44802 (44.6)	0.01
Congenital malformation [Q00-99]	6954 (6.9)	6767 (6.7)	0.0074
Injury/poisoning [S00-T88]	30848 (30.7)	30588 (30.4)	0.0056
Falls [W00-19]	4998 (5.0)	4906 (4.9)	0.0042
**FACTORS AFFECTING HEALTH**			
History of coded normal general examination	36008 (35.8)	35630 (35.4)	0.0079
History of coded abnormal general examination	643 (0.64)	660 (0.66)	0.0021
History of cancer screening	34993 (34.8)	34821 (34.6)	0.0036
Long-term drug therapy	18474 (18.4)	18304 (18.2)	0.0044
History of nicotine dependence	7826 (7.8)	7809 (7.8)	0.00063
Prior influenza vaccine	42033 (41.8)	41461 (41.2)	0.012

Supplementary Table 6 Baseline characteristics for the analysis based on restricted exposure windows. SMD=Standardised mean difference.

	**Nov 17-Apr 18**	**Apr 17- Sep 17**	**SMD**
**Number**	20243	20243	-
**SOCIODEMOGRAPHICS**			
Age; mean (SD); y	70.9 (4.9)	71.0 (4.9)	0.018
Sex; n (%)			
Female	10989 (54.3)	10962 (54.2)	0.0027
Male	8422 (41.6)	8425 (41.6)	3e-04
Other	832 (4.1)	856 (4.2)	0.0059
Race; n (%)			
White	14731 (72.8)	14654 (72.4)	0.0085
Black or African American	2045 (10.1)	2137 (10.6)	0.015
Asian	874 (4.3)	846 (4.2)	0.0069
Other	452 (2.2)	450 (2.2)	0.00067
Unknown	1996 (9.9)	2012 (9.9)	0.0026
Ethnicity; n (%)			
Hispanic or Latino	1321 (6.5)	1334 (6.6)	0.0026
Not Hispanic of Latino	15661 (77.4)	15666 (77.4)	0.00059
Unknown	3261 (16.1)	3243 (16.0)	0.0024
Marital status; n (%)			
Married	5578 (27.6)	5448 (26.9)	0.014
Never married	1532 (7.6)	1498 (7.4)	0.0064
Divorced	1269 (6.3)	1311 (6.5)	0.0085
Domestic partner	11 (0.054)	14 (0.069)	0.006
**COMORBIDITIES [ICD-10 code]; n (%)**			
Infections and parasites [A00-B99]	5700 (28.2)	5645 (27.9)	0.006
Herpes simplex infection [B00]	374 (1.8)	365 (1.8)	0.0033
Herpes zoster infection [B02]	929 (4.6)	941 (4.6)	0.0028
Neoplasms (benign and malignant) [C00-D49]	8010 (39.6)	7934 (39.2)	0.0077
Skin cancer [C43-44]	1264 (6.2)	1265 (6.2)	2e-04
In situ neoplasms [D00-09]	605 (3.0)	605 (3.0)	0
Benign neoplasms [D10-36]	5425 (26.8)	5361 (26.5)	0.0072
Neoplasms of uncertain behaviour [D37-38]	2256 (11.1)	2276 (11.2)	0.0031
Haematological conditions [D50-89]	4813 (23.8)	4802 (23.7)	0.0013
Thyroid disorders [E00-07]	4253 (21.0)	4156 (20.5)	0.012
Iodine-deficiency disorder [E01]	649 (3.2)	675 (3.3)	0.0072
Hypothyroidism [E03]	3371 (16.7)	3280 (16.2)	0.012
Nontoxic goiter [E04]	1009 (5.0)	1007 (5.0)	0.00045
Hyperthyroidism [E05]	380 (1.9)	378 (1.9)	0.00073
Other disorders of thyroid [E07]	618 (3.1)	639 (3.2)	0.006
Diabetes mellitus [E08-13]	5264 (26.0)	5232 (25.8)	0.0036
Vitamin B deficiency [E53]	928 (4.6)	942 (4.7)	0.0033

Vitamin D deficiency [E55]	3790 (18.7)	3801 (18.8)	0.0014
Overweight and obesity [E66]	4072 (20.1)	4043 (20.0)	0.0036
Metabolic disorders [E70-88]	13170 (65.1)	13068 (64.6)	0.011
Psychiatric comorbidities			
Substance use disorder [F10-19]	2515 (12.4)	2479 (12.2)	0.0054
Mood disorder [F30-39]	3702 (18.3)	3649 (18.0)	0.0068
Anxiety disorder [F40-48]	3949 (19.5)	3926 (19.4)	0.0029
Organic disorder [F50-59]	977 (4.8)	994 (4.9)	0.0039
Neurological disorder [G00-99]	9427 (46.6)	9342 (46.1)	0.0084
Ophthalmic disorder [H00-59]	6936 (34.3)	6907 (34.1)	0.003
Ear/mastoid process disease [H60-95]	5226 (25.8)	5162 (25.5)	0.0072
Cardiovascular disease [I00-99]	14416 (71.2)	14308 (70.7)	0.012
Chronic rheumatic heart diseases [I05-09]	957 (4.7)	953 (4.7)	0.00093
Hypertension [I10]	12516 (61.8)	12421 (61.4)	0.0096
Ischaemic heart disease [I20-25]	3620 (17.9)	3615 (17.9)	0.00064
Other heart disease [I30-52]	5511 (27.2)	5500 (27.2)	0.0012
Cerebrovascular diseases [I60-69]	1881 (9.3)	1909 (9.4)	0.0047
Cerebral infarction [I63]	708 (3.5)	724 (3.6)	0.0043
Atherosclerosis [I70]	1037 (5.1)	1027 (5.1)	0.0022
Other peripheral vascular diseases [I73]	1165 (5.8)	1170 (5.8)	0.0011
Other disorders of arteries/arterioles [I77]	697 (3.4)	700 (3.5)	0.00081
Disease of veins/lymphatics [I80-89]	2047 (10.1)	2049 (10.1)	0.00033
Unspecified disease of circulatory system [I95-99]	1263 (6.2)	1249 (6.2)	0.0029
Respiratory disease [J00-99]	9876 (48.8)	9795 (48.4)	0.008
Digestive disease [K00-95]	10954 (54.1)	10844 (53.6)	0.011
Dermatological disease [L00-99]	8491 (41.9)	8437 (41.7)	0.0054
Musculoskeletal disease [M00-99]	13967 (69.0)	13832 (68.3)	0.014
Genitourinary disease [N00-99]	10462 (51.7)	10387 (51.3)	0.0074
Congenital malformation [Q00-99]	1724 (8.5)	1672 (8.3)	0.0093
Injury/poisoning [S00-T88]	7670 (37.9)	7564 (37.4)	0.011
Falls [W00-19]	1220 (6.0)	1230 (6.1)	0.0021
**FACTORS AFFECTING HEALTH**			
History of coded normal general examination	8373 (41.4)	8319 (41.1)	0.0054
History of coded abnormal general examination	337 (1.7)	341 (1.7)	0.0015
History of cancer screening	8558 (42.3)	8506 (42.0)	0.0052
Long-term drug therapy	4697 (23.2)	4669 (23.1)	0.0033
History of nicotine dependence	2395 (11.8)	2392 (11.8)	0.00046
Prior influenza vaccine	9060 (44.8)	9071 (44.8)	0.0011

Supplementary Table 7 Baseline characteristics for the analysis restricted to females. SMD=Standardised mean difference.

	After Oct 2017	Before Oct 2017	SMD
**Number**	54846	54846	-
**SOCIODEMOGRAPHICS**			
Age; mean (SD); y	70.7 (4.8)	70.8 (4.9)	0.011
Sex; n (%)			
Female	54846 (100.0)	54846 (100.0)	NA
Male	0 (0.0)	0 (0.0)	NA
Other/Unknown	0 (0.0)	0 (0.0)	NA
Race; n (%)			
White	40246 (73.4)	39922 (72.8)	0.013
Black or African American	6801 (12.4)	6814 (12.4)	0.00072
Asian	2107 (3.8)	2121 (3.9)	0.0013
Other	991 (1.8)	1130 (2.1)	0.018
Unknown	4332 (7.9)	4502 (8.2)	0.011
Ethnicity; n (%)			
Hispanic or Latino	2982 (5.4)	3063 (5.6)	0.0065
Not Hispanic of Latino	43416 (79.2)	42491 (77.5)	0.041
Unknown	8448 (15.4)	9292 (16.9)	0.042
Marital status; n (%)			
Married	14142 (25.8)	14066 (25.6)	0.0032
Never married	3976 (7.2)	4026 (7.3)	0.0035
Divorced	4111 (7.5)	4138 (7.5)	0.0019
Domestic partner	38 (0.069)	33 (0.06)	0.0036
**COMORBIDITIES [ICD-10 code]; n (%)**			
Infections and parasites [A00-B99]	13605 (24.8)	13350 (24.3)	0.011
Herpes simplex infection [B00]	907 (1.7)	910 (1.7)	0.00043
Herpes zoster infection [B02]	2603 (4.7)	2582 (4.7)	0.0018
Neoplasms (benign and malignant) [C00-D49]	18051 (32.9)	17868 (32.6)	0.0071
Skin cancer [C43-44]	2153 (3.9)	2161 (3.9)	0.00075
In situ neoplasms [D00-09]	1398 (2.5)	1425 (2.6)	0.0031
Benign neoplasms [D10-36]	12018 (21.9)	11906 (21.7)	0.0049
Neoplasms of uncertain behaviour [D37-38]	4046 (7.4)	4046 (7.4)	0
Haematological conditions [D50-89]	11228 (20.5)	11174 (20.4)	0.0024
Thyroid disorders [E00-07]	13199 (24.1)	13027 (23.8)	0.0074
Iodine-deficiency disorder [E01]	1864 (3.4)	1818 (3.3)	0.0047
Hypothyroidism [E03]	10414 (19.0)	10294 (18.8)	0.0056
Nontoxic goiter [E04]	3307 (6.0)	3225 (5.9)	0.0063
Hyperthyroidism [E05]	1366 (2.5)	1297 (2.4)	0.0082
Other disorders of thyroid [E07]	1787 (3.3)	1722 (3.1)	0.0067
Diabetes mellitus [E08-13]	12604 (23.0)	12495 (22.8)	0.0047

Vitamin B deficiency [E53]	2160 (3.9)	2166 (3.9)	0.00056
Vitamin D deficiency [E55]	10078 (18.4)	10129 (18.5)	0.0024
Overweight and obesity [E66]	9881 (18.0)	9727 (17.7)	0.0073
Metabolic disorders [E70-88]	31825 (58.0)	31471 (57.4)	0.013
Psychiatric comorbidities			
Substance use disorder [F10-19]	4926 (9.0)	4849 (8.8)	0.0049
Mood disorder [F30-39]	10979 (20.0)	10827 (19.7)	0.0069
Anxiety disorder [F40-48]	10947 (20.0)	10896 (19.9)	0.0023
Organic disorder [F50-59]	1237 (2.3)	1223 (2.2)	0.0017
Neurological disorder [G00-99]	22873 (41.7)	22677 (41.3)	0.0073
Ophthalmic disorder [H00-59]	17251 (31.5)	17187 (31.3)	0.0025
Ear/mastoid process disease [H60-95]	11918 (21.7)	11688 (21.3)	0.01
Cardiovascular disease [I00-99]	35033 (63.9)	34672 (63.2)	0.014
Chronic rheumatic heart diseases [I05-09]	1817 (3.3)	1713 (3.1)	0.011
Hypertension [I10]	30686 (55.9)	30270 (55.2)	0.015
Ischaemic heart disease [I20-25]	6605 (12.0)	6483 (11.8)	0.0069
Other heart disease [I30-52]	12100 (22.1)	11996 (21.9)	0.0046
Cerebrovascular diseases [I60-69]	4570 (8.3)	4527 (8.3)	0.0028
Cerebral infarction [I63]	1623 (3.0)	1591 (2.9)	0.0035
Atherosclerosis [I70]	2225 (4.1)	2235 (4.1)	0.00092
Other peripheral vascular diseases [I73]	2581 (4.7)	2544 (4.6)	0.0032
Other disorders of arteries/arterioles [I77]	1240 (2.3)	1216 (2.2)	0.003
Disease of veins/lymphatics [I80-89]	5255 (9.6)	5185 (9.5)	0.0043
Unspecified disease of circulatory system [I95-99]	2882 (5.3)	2809 (5.1)	0.006
Respiratory disease [J00-99]	24819 (45.3)	24552 (44.8)	0.0098
Digestive disease [K00-95]	26930 (49.1)	26668 (48.6)	0.0096
Dermatological disease [L00-99]	20059 (36.6)	19764 (36.0)	0.011
Musculoskeletal disease [M00-99]	36652 (66.8)	36243 (66.1)	0.016
Genitourinary disease [N00-99]	24182 (44.1)	23995 (43.8)	0.0069
Congenital malformation [Q00-99]	3969 (7.2)	3976 (7.2)	0.00049
Injury/poisoning [S00-T88]	17844 (32.5)	17703 (32.3)	0.0055
Falls [W00-19]	3412 (6.2)	3321 (6.1)	0.0069
**FACTORS AFFECTING HEALTH**			
History of coded normal general examination	18860 (34.4)	18622 (34.0)	0.0091
History of coded abnormal general examination	315 (0.57)	333 (0.61)	0.0043
History of cancer screening	21384 (39.0)	21091 (38.5)	0.011
Long-term drug therapy	10391 (18.9)	10222 (18.6)	0.0079
History of nicotine dependence	3291 (6.0)	3284 (6.0)	0.00054
Prior influenza vaccine	23570 (43.0)	23321 (42.5)	0.0092

Supplementary Table 8 Baseline characteristics for the analysis restricted to males. SMD=Standardised mean difference.

	After Oct 2017	Before Oct 2017	SMD
**Number**	43990	43990	-
**SOCIODEMOGRAPHICS**			
Age; mean (SD); y	70.6 (4.7)	70.6 (4.7)	0.014
Sex; n (%)			
Female	0 (0.0)	0 (0.0)	NA
Male	43990 (100.0)	43990 (100.0)	NA
Other/Unknown	0 (0.0)	0 (0.0)	NA
Race; n (%)			
White	34352 (78.1)	34048 (77.4)	0.017
Black or African American	3989 (9.1)	3927 (8.9)	0.0049
Asian	1637 (3.7)	1721 (3.9)	0.01
Other	763 (1.7)	898 (2.0)	0.023
Unknown	2959 (6.7)	3124 (7.1)	0.015
Ethnicity; n (%)			
Hispanic or Latino	1956 (4.4)	2006 (4.6)	0.0055
Not Hispanic of Latino	35503 (80.7)	34836 (79.2)	0.038
Unknown	6531 (14.8)	7148 (16.2)	0.039
Marital status; n (%)			
Married	17327 (39.4)	17459 (39.7)	0.0061
Never married	2770 (6.3)	2797 (6.4)	0.0025
Divorced	1847 (4.2)	1782 (4.1)	0.0074
Domestic partner	47 (0.11)	41 (0.093)	0.0043
**COMORBIDITIES [ICD-10 code]; n (%)**			
Infections and parasites [A00-B99]	9724 (22.1)	9597 (21.8)	0.007
Herpes simplex infection [B00]	429 (0.97)	407 (0.93)	0.0052
Herpes zoster infection [B02]	1539 (3.5)	1477 (3.4)	0.0077
Neoplasms (benign and malignant) [C00-D49]	15299 (34.8)	15210 (34.6)	0.0043
Skin cancer [C43-44]	2997 (6.8)	2911 (6.6)	0.0078
In situ neoplasms [D00-09]	1012 (2.3)	1013 (2.3)	0.00015
Benign neoplasms [D10-36]	9461 (21.5)	9447 (21.5)	0.00077
Neoplasms of uncertain behaviour [D37-38]	3789 (8.6)	3778 (8.6)	0.00089
Haematological conditions [D50-89]	8534 (19.4)	8381 (19.1)	0.0088
Thyroid disorders [E00-07]	4154 (9.4)	4193 (9.5)	0.003
Iodine-deficiency disorder [E01]	500 (1.1)	493 (1.1)	0.0015
Hypothyroidism [E03]	3251 (7.4)	3257 (7.4)	0.00052
Nontoxic goiter [E04]	831 (1.9)	834 (1.9)	5e-04
Hyperthyroidism [E05]	408 (0.93)	399 (0.91)	0.0021
Other disorders of thyroid [E07]	468 (1.1)	505 (1.1)	0.008
Diabetes mellitus [E08-13]	10968 (24.9)	10871 (24.7)	0.0051
Vitamin B deficiency [E53]	1135 (2.6)	1135 (2.6)	0

Vitamin D deficiency [E55]	4347 (9.9)	4313 (9.8)	0.0026
Overweight and obesity [E66]	6604 (15.0)	6515 (14.8)	0.0057
Metabolic disorders [E70-88]	25534 (58.0)	25284 (57.5)	0.012
Psychiatric comorbidities			
Substance use disorder [F10-19]	5453 (12.4)	5405 (12.3)	0.0033
Mood disorder [F30-39]	4521 (10.3)	4559 (10.4)	0.0028
Anxiety disorder [F40-48]	4672 (10.6)	4628 (10.5)	0.0033
Organic disorder [F50-59]	1768 (4.0)	1716 (3.9)	0.0061
Neurological disorder [G00-99]	16354 (37.2)	16234 (36.9)	0.0056
Ophthalmic disorder [H00-59]	12145 (27.6)	12070 (27.4)	0.0038
Ear/mastoid process disease [H60-95]	9174 (20.9)	9141 (20.8)	0.0018
Cardiovascular disease [I00-99]	28943 (65.8)	28653 (65.1)	0.014
Chronic rheumatic heart diseases [I05-09]	1222 (2.8)	1207 (2.7)	0.0021
Hypertension [I10]	24619 (56.0)	24373 (55.4)	0.011
Ischaemic heart disease [I20-25]	9437 (21.5)	9296 (21.1)	0.0078
Other heart disease [I30-52]	11346 (25.8)	11269 (25.6)	0.004
Cerebrovascular diseases [I60-69]	4004 (9.1)	3919 (8.9)	0.0068
Cerebral infarction [I63]	1538 (3.5)	1542 (3.5)	0.00049
Atherosclerosis [I70]	2295 (5.2)	2270 (5.2)	0.0026
Other peripheral vascular diseases [I73]	2556 (5.8)	2502 (5.7)	0.0053
Other disorders of arteries/arterioles [I77]	1312 (3.0)	1339 (3.0)	0.0036
Disease of veins/lymphatics [I80-89]	3405 (7.7)	3354 (7.6)	0.0044
Unspecified disease of circulatory system [I95-99]	2520 (5.7)	2458 (5.6)	0.0061
Respiratory disease [J00-99]	17356 (39.5)	17212 (39.1)	0.0067
Digestive disease [K00-95]	20272 (46.1)	20165 (45.8)	0.0049
Dermatological disease [L00-99]	15232 (34.6)	14979 (34.1)	0.012
Musculoskeletal disease [M00-99]	24388 (55.4)	24197 (55.0)	0.0087
Genitourinary disease [N00-99]	20335 (46.2)	20095 (45.7)	0.011
Congenital malformation [Q00-99]	2891 (6.6)	2885 (6.6)	0.00055
Injury/poisoning [S00-T88]	13119 (29.8)	12914 (29.4)	0.01
Falls [W00-19]	1648 (3.7)	1584 (3.6)	0.0077
**FACTORS AFFECTING HEALTH**			
History of coded normal general examination	14867 (33.8)	14750 (33.5)	0.0056
History of coded abnormal general examination	330 (0.75)	320 (0.73)	0.0027
History of cancer screening	12283 (27.9)	12030 (27.3)	0.013
Long-term drug therapy	7901 (18.0)	7796 (17.7)	0.0062
History of nicotine dependence	4271 (9.7)	4269 (9.7)	0.00015
Prior influenza vaccine	19227 (43.7)	19009 (43.2)	0.01

Supplementary Table 9 Distribution of the core set of covariates after coarsened exact matching.

	**Exact matched (before Oct 2017)**	**Exact matched (after Oct 2017)**
Sample size	82102	82102
Age category		
65-67	31742	31742
68-70	22554	22554
71-73	15847	15847
74-76	11959	11959
Sex		
Male	36932	36932
Female	45170	45170
Race		
White	62407	62407
Non-white	19695	19695
Neurological comorbidity		
Yes	28426	28426
No	53676	53676

Supplementary Table 10 Summary results for the analysis between shingles vaccines and two other commonly used vaccines.

	N	RMTL	p	Additional time lived diagnosis-free among affected people, days (95% CI)
**Recombinant shingles vs Influenza vaccine**	209031	0.77 (0.75-0.79)	1.4e-67	213 (189-237)
**Recombinant shingles vs Tdap vaccine**	98353	0.73 (0.70-0.76)	2.6e-53	271 (237-306)
**Live shingles vs Influenza vaccine**	41466	0.86 (0.81-0.91)	1.2e-06	144 (85-202)
**Live shingles vs Tdap vaccine**	64035	0.86 (0.82-0.90)	2.1e-09	142 (96-189)

The p-values correspond to the z-test defined in the SurvRM2 package in R, two-sided and not corrected for multiple comparisons.

Supplementary Table 11 Baseline characteristics for the comparison between recombinant zoster vaccine and influenza vaccine. SMD=Standardised mean difference.

	Recombinant	Influenza vaccine	SMD
**Number**	209031	209031	-
**SOCIODEMOGRAPHICS**			
Age; mean (SD); y	72.2 (5.5)	72.1 (5.4)	0.011
Sex; n (%)			
Female	117278 (56.1)	118219 (56.6)	0.0091
Male	90463 (43.3)	89637 (42.9)	0.008
Other/Unknown	1290 (0.62)	1175 (0.56)	0.0072
Race; n (%)			
White	165449 (79.2)	166885 (79.8)	0.017
Black or African American	16941 (8.1)	15904 (7.6)	0.018
Asian	11881 (5.7)	11619 (5.6)	0.0054
Other	4416 (2.1)	4574 (2.2)	0.0052
Unknown	9016 (4.3)	8771 (4.2)	0.0058
Ethnicity; n (%)			
Hispanic or Latino	8332 (4.0)	8037 (3.8)	0.0073
Not Hispanic of Latino	184263 (88.2)	184709 (88.4)	0.0066
Unknown	16436 (7.9)	16285 (7.8)	0.0027
Marital status; n (%)			
Married	66023 (31.6)	61807 (29.6)	0.044
Never married	12785 (6.1)	11729 (5.6)	0.022
Divorced	9946 (4.8)	9315 (4.5)	0.014
Domestic partner	202 (0.097)	184 (0.088)	0.0028
**COMORBIDITIES [ICD-10 code]; n (%)**			
Infections and parasites [A00-B99]	65313 (31.2)	63513 (30.4)	0.019
Herpes simplex infection [B00]	5075 (2.4)	4801 (2.3)	0.0086
Herpes zoster infection [B02]	9700 (4.6)	9506 (4.5)	0.0044
Neoplasms (benign and malignant) [C00-D49]	95400 (45.6)	94510 (45.2)	0.0086
Skin cancer [C43-44]	17340 (8.3)	17175 (8.2)	0.0029
In situ neoplasms [D00-09]	8204 (3.9)	8281 (4.0)	0.0019
Benign neoplasms [D10-36]	63848 (30.5)	63376 (30.3)	0.0049
Neoplasms of uncertain behaviour [D37-38]	24424 (11.7)	24485 (11.7)	0.00091
Haematological conditions [D50-89]	58887 (28.2)	57322 (27.4)	0.017
Thyroid disorders [E00-07]	48889 (23.4)	48472 (23.2)	0.0047
Iodine-deficiency disorder [E01]	3242 (1.6)	3280 (1.6)	0.0015
Hypothyroidism [E03]	37891 (18.1)	37540 (18.0)	0.0044
Nontoxic goiter [E04]	12628 (6.0)	12585 (6.0)	0.00086
Hyperthyroidism [E05]	4494 (2.1)	4499 (2.2)	0.00016
Other disorders of thyroid [E07]	6314 (3.0)	6200 (3.0)	0.0032
Diabetes mellitus [E08-13]	50995 (24.4)	49848 (23.8)	0.013

Vitamin B deficiency [E53]	10742 (5.1)	10524 (5.0)	0.0047
Vitamin D deficiency [E55]	43949 (21.0)	43722 (20.9)	0.0027
Overweight and obesity [E66]	49587 (23.7)	48411 (23.2)	0.013
Metabolic disorders [E70-88]	141536 (67.7)	138709 (66.4)	0.029
Psychiatric comorbidities			
Substance use disorder [F10-19]	23466 (11.2)	22607 (10.8)	0.013
Mood disorder [F30-39]	40773 (19.5)	40141 (19.2)	0.0077
Anxiety disorder [F40-48]	45857 (21.9)	45127 (21.6)	0.0085
Organic disorder [F50-59]	11160 (5.3)	11083 (5.3)	0.0016
Neurological disorder [G00-99]	106585 (51.0)	104697 (50.1)	0.018
Ophthalmic disorder [H00-59]	80535 (38.5)	80632 (38.6)	0.00095
Ear/mastoid process disease [H60-95]	60311 (28.9)	59117 (28.3)	0.013
Cardiovascular disease [I00-99]	149274 (71.4)	146171 (69.9)	0.033
Chronic rheumatic heart diseases [I05-09]	8311 (4.0)	8067 (3.9)	0.006
Hypertension [I10]	126751 (60.6)	124882 (59.7)	0.018
Ischaemic heart disease [I20-25]	40765 (19.5)	39469 (18.9)	0.016
Other heart disease [I30-52]	64467 (30.8)	62365 (29.8)	0.022
Cerebrovascular diseases [I60-69]	21080 (10.1)	20395 (9.8)	0.011
Cerebral infarction [I63]	7432 (3.6)	7147 (3.4)	0.0074
Atherosclerosis [I70]	14340 (6.9)	14082 (6.7)	0.0049
Other peripheral vascular diseases [I73]	13705 (6.6)	13528 (6.5)	0.0034
Other disorders of arteries/arterioles [I77]	9768 (4.7)	9665 (4.6)	0.0023
Disease of veins/lymphatics [I80-89]	25245 (12.1)	24788 (11.9)	0.0067
Unspecified disease of circulatory system [I95-99]	16045 (7.7)	15282 (7.3)	0.014
Respiratory disease [J00-99]	108140 (51.7)	105604 (50.5)	0.024
Digestive disease [K00-95]	124366 (59.5)	121905 (58.3)	0.024
Dermatological disease [L00-99]	95997 (45.9)	94744 (45.3)	0.012
Musculoskeletal disease [M00-99]	152592 (73.0)	150938 (72.2)	0.018
Genitourinary disease [N00-99]	116639 (55.8)	114300 (54.7)	0.023
Congenital malformation [Q00-99]	19003 (9.1)	18856 (9.0)	0.0025
Injury/poisoning [S00-T88]	86955 (41.6)	84455 (40.4)	0.024
Falls [W00-19]	16979 (8.1)	16083 (7.7)	0.016
**FACTORS AFFECTING HEALTH**			
History of coded normal general examination	85026 (40.7)	82136 (39.3)	0.028
History of coded abnormal general examination	6802 (3.3)	5492 (2.6)	0.037
History of cancer screening	86680 (41.5)	84217 (40.3)	0.024
Long-term drug therapy	67300 (32.2)	64271 (30.7)	0.031
History of nicotine dependence	34201 (16.4)	32209 (15.4)	0.026
Prior influenza vaccine	209031 (100.0)	209031 (100.0)	NA

Supplementary Table 12 Baseline characteristics for the comparison between recombinant zoster vaccine and Tdap vaccine. SMD=Standardised mean difference.

	Recombinant	Tdap vaccine	SMD
**Number**	98353	98353	-
**SOCIODEMOGRAPHICS**			
Age; mean (SD); y	72.4 (5.7)	72.4 (5.7)	8e-04
Sex; n (%)			
Female	50556 (51.4)	50680 (51.5)	0.0025
Male	44742 (45.5)	44841 (45.6)	0.002
Other/Unknown	3055 (3.1)	2832 (2.9)	0.013
Race; n (%)			
White	73506 (74.7)	73803 (75.0)	0.007
Black or African American	9183 (9.3)	9086 (9.2)	0.0034
Asian	3899 (4.0)	3854 (3.9)	0.0024
Other	2557 (2.6)	2599 (2.6)	0.0027
Unknown	8672 (8.8)	8479 (8.6)	0.007
Ethnicity; n (%)			
Hispanic or Latino	4522 (4.6)	4299 (4.4)	0.011
Not Hispanic of Latino	73848 (75.1)	74274 (75.5)	0.01
Unknown	19983 (20.3)	19780 (20.1)	0.0051
Marital status; n (%)			
Married	28676 (29.2)	27724 (28.2)	0.021
Never married	6441 (6.5)	6141 (6.2)	0.012
Divorced	4981 (5.1)	4813 (4.9)	0.0079
Domestic partner	89 (0.09)	83 (0.084)	0.0021
**COMORBIDITIES [ICD-10 code]; n (%)**			
Infections and parasites [A00-B99]	25809 (26.2)	25720 (26.2)	0.0021
Herpes simplex infection [B00]	1664 (1.7)	1523 (1.5)	0.011
Herpes zoster infection [B02]	3431 (3.5)	3138 (3.2)	0.017
Neoplasms (benign and malignant) [C00-D49]	36410 (37.0)	35892 (36.5)	0.011
Skin cancer [C43-44]	6475 (6.6)	6296 (6.4)	0.0074
In situ neoplasms [D00-09]	3137 (3.2)	3086 (3.1)	0.003
Benign neoplasms [D10-36]	23267 (23.7)	22413 (22.8)	0.021
Neoplasms of uncertain behaviour [D37-38]	9134 (9.3)	9002 (9.2)	0.0046
Haematological conditions [D50-89]	25643 (26.1)	25784 (26.2)	0.0033
Thyroid disorders [E00-07]	19675 (20.0)	19064 (19.4)	0.016
Iodine-deficiency disorder [E01]	1662 (1.7)	1547 (1.6)	0.0092
Hypothyroidism [E03]	15394 (15.7)	14807 (15.1)	0.017
Nontoxic goiter [E04]	4734 (4.8)	4668 (4.7)	0.0031
Hyperthyroidism [E05]	1776 (1.8)	1747 (1.8)	0.0022
Other disorders of thyroid [E07]	2655 (2.7)	2565 (2.6)	0.0057
Diabetes mellitus [E08-13]	22214 (22.6)	21848 (22.2)	0.0089
Vitamin B deficiency [E53]	4103 (4.2)	4037 (4.1)	0.0034

Vitamin D deficiency [E55]	15883 (16.1)	15187 (15.4)	0.019
Overweight and obesity [E66]	17902 (18.2)	17485 (17.8)	0.011
Metabolic disorders [E70-88]	58140 (59.1)	56981 (57.9)	0.024
Psychiatric comorbidities			
Substance use disorder [F10-19]	11987 (12.2)	11804 (12.0)	0.0057
Mood disorder [F30-39]	16499 (16.8)	16455 (16.7)	0.0012
Anxiety disorder [F40-48]	18069 (18.4)	17908 (18.2)	0.0042
Organic disorder [F50-59]	4010 (4.1)	3848 (3.9)	0.0084
Neurological disorder [G00-99]	43987 (44.7)	43369 (44.1)	0.013
Ophthalmic disorder [H00-59]	29426 (29.9)	29040 (29.5)	0.0086
Ear/mastoid process disease [H60-95]	21902 (22.3)	21229 (21.6)	0.017
Cardiovascular disease [I00-99]	65346 (66.4)	63558 (64.6)	0.038
Chronic rheumatic heart diseases [I05-09]	3949 (4.0)	4042 (4.1)	0.0048
Hypertension [I10]	54763 (55.7)	53306 (54.2)	0.03
Ischaemic heart disease [I20-25]	18581 (18.9)	18835 (19.1)	0.0066
Other heart disease [I30-52]	29195 (29.7)	29149 (29.6)	0.001
Cerebrovascular diseases [I60-69]	10537 (10.7)	10576 (10.8)	0.0013
Cerebral infarction [I63]	3760 (3.8)	3842 (3.9)	0.0043
Atherosclerosis [I70]	6177 (6.3)	6369 (6.5)	0.008
Other peripheral vascular diseases [I73]	5807 (5.9)	6053 (6.2)	0.011
Other disorders of arteries/arterioles [I77]	4241 (4.3)	4359 (4.4)	0.0059
Disease of veins/lymphatics [I80-89]	10649 (10.8)	10864 (11.0)	0.007
Unspecified disease of circulatory system [I95-99]	7623 (7.8)	7877 (8.0)	0.0096
Respiratory disease [J00-99]	44351 (45.1)	43708 (44.4)	0.013
Digestive disease [K00-95]	50219 (51.1)	49583 (50.4)	0.013
Dermatological disease [L00-99]	37635 (38.3)	37138 (37.8)	0.01
Musculoskeletal disease [M00-99]	63755 (64.8)	62342 (63.4)	0.03
Genitourinary disease [N00-99]	47418 (48.2)	46846 (47.6)	0.012
Congenital malformation [Q00-99]	7155 (7.3)	7130 (7.2)	0.00098
Injury/poisoning [S00-T88]	49375 (50.2)	47276 (48.1)	0.043
Falls [W00-19]	12036 (12.2)	10032 (10.2)	0.065
**FACTORS AFFECTING HEALTH**			
History of coded normal general examination	30753 (31.3)	28544 (29.0)	0.049
History of coded abnormal general examination	2211 (2.2)	1744 (1.8)	0.034
History of cancer screening	32468 (33.0)	30779 (31.3)	0.037
Long-term drug therapy	28892 (29.4)	27619 (28.1)	0.029
History of nicotine dependence	14076 (14.3)	13671 (13.9)	0.012
Prior influenza vaccine	41734 (42.4)	40612 (41.3)	0.023

Supplementary Table 13 Baseline characteristics for the comparison between live zoster vaccine and influenza vaccine. SMD=Standardised mean difference.

	**Live**	**Influenza vaccine**	**SMD**
**Number**	41466	41466	-
**SOCIODEMOGRAPHICS**			
Age; mean (SD); y	71.0 (4.8)	70.8 (4.8)	0.031
Sex; n (%)			
Female	22709 (54.8)	22686 (54.7)	0.0011
Male	18539 (44.7)	18580 (44.8)	0.002
Other/Unknown	218 (0.53)	200 (0.48)	0.0061
Race; n (%)			
White	30127 (72.7)	30643 (73.9)	0.028
Black or African American	6053 (14.6)	5808 (14.0)	0.017
Asian	1924 (4.6)	1896 (4.6)	0.0032
Other	975 (2.4)	941 (2.3)	0.0055
Unknown	2024 (4.9)	1828 (4.4)	0.022
Ethnicity; n (%)			
Hispanic or Latino	2298 (5.5)	2124 (5.1)	0.019
Not Hispanic of Latino	35217 (84.9)	35605 (85.9)	0.026
Unknown	3951 (9.5)	3737 (9.0)	0.018
Marital status; n (%)			
Married	11627 (28.0)	11068 (26.7)	0.03
Never married	2904 (7.0)	2677 (6.5)	0.022
Divorced	2371 (5.7)	2164 (5.2)	0.022
Domestic partner	22 (0.053)	25 (0.06)	0.003
**COMORBIDITIES [ICD-10 code]; n (%)**			
Infections and parasites [A00-B99]	10709 (25.8)	10265 (24.8)	0.025
Herpes simplex infection [B00]	584 (1.4)	552 (1.3)	0.0066
Herpes zoster infection [B02]	1713 (4.1)	1547 (3.7)	0.021
Neoplasms (benign and malignant) [C00-D49]	14715 (35.5)	14403 (34.7)	0.016
Skin cancer [C43-44]	2142 (5.2)	2164 (5.2)	0.0024
In situ neoplasms [D00-09]	1017 (2.5)	958 (2.3)	0.0093
Benign neoplasms [D10-36]	9450 (22.8)	9315 (22.5)	0.0078
Neoplasms of uncertain behaviour [D37-38]	3539 (8.5)	3467 (8.4)	0.0062
Haematological conditions [D50-89]	9366 (22.6)	9038 (21.8)	0.019
Thyroid disorders [E00-07]	7315 (17.6)	7289 (17.6)	0.0016
Iodine-deficiency disorder [E01]	780 (1.9)	799 (1.9)	0.0034
Hypothyroidism [E03]	5625 (13.6)	5619 (13.6)	0.00042
Nontoxic goiter [E04]	1875 (4.5)	1814 (4.4)	0.0071
Hyperthyroidism [E05]	801 (1.9)	800 (1.9)	0.00018
Other disorders of thyroid [E07]	958 (2.3)	961 (2.3)	0.00048
Diabetes mellitus [E08-13]	11417 (27.5)	11057 (26.7)	0.02
Vitamin B deficiency [E53]	1435 (3.5)	1410 (3.4)	0.0033

Vitamin D deficiency [E55]	6456 (15.6)	6246 (15.1)	0.014
Overweight and obesity [E66]	7619 (18.4)	7243 (17.5)	0.024
Metabolic disorders [E70-88]	24081 (58.1)	23432 (56.5)	0.032
Psychiatric comorbidities			
Substance use disorder [F10-19]	4845 (11.7)	4555 (11.0)	0.022
Mood disorder [F30-39]	7201 (17.4)	6893 (16.6)	0.02
Anxiety disorder [F40-48]	6889 (16.6)	6600 (15.9)	0.019
Organic disorder [F50-59]	1403 (3.4)	1354 (3.3)	0.0066
Neurological disorder [G00-99]	17093 (41.2)	16384 (39.5)	0.035
Ophthalmic disorder [H00-59]	13911 (33.5)	13601 (32.8)	0.016
Ear/mastoid process disease [H60-95]	9158 (22.1)	8713 (21.0)	0.026
Cardiovascular disease [I00-99]	27371 (66.0)	26606 (64.2)	0.039
Chronic rheumatic heart diseases [I05-09]	1417 (3.4)	1341 (3.2)	0.01
Hypertension [I10]	23664 (57.1)	23024 (55.5)	0.031
Ischaemic heart disease [I20-25]	7541 (18.2)	7167 (17.3)	0.024
Other heart disease [I30-52]	10729 (25.9)	10334 (24.9)	0.022
Cerebrovascular diseases [I60-69]	3965 (9.6)	3761 (9.1)	0.017
Cerebral infarction [I63]	1569 (3.8)	1421 (3.4)	0.019
Atherosclerosis [I70]	2240 (5.4)	2195 (5.3)	0.0048
Other peripheral vascular diseases [I73]	2566 (6.2)	2480 (6.0)	0.0087
Other disorders of arteries/arterioles [I77]	1236 (3.0)	1195 (2.9)	0.0059
Disease of veins/lymphatics [I80-89]	4066 (9.8)	4016 (9.7)	0.0041
Unspecified disease of circulatory system [I95-99]	2779 (6.7)	2651 (6.4)	0.012
Respiratory disease [J00-99]	18054 (43.5)	17385 (41.9)	0.033
Digestive disease [K00-95]	20783 (50.1)	20038 (48.3)	0.036
Dermatological disease [L00-99]	15298 (36.9)	14881 (35.9)	0.021
Musculoskeletal disease [M00-99]	26020 (62.8)	25550 (61.6)	0.023
Genitourinary disease [N00-99]	19349 (46.7)	18692 (45.1)	0.032
Congenital malformation [Q00-99]	3282 (7.9)	3124 (7.5)	0.014
Injury/poisoning [S00-T88]	13636 (32.9)	13062 (31.5)	0.03
Falls [W00-19]	2639 (6.4)	2489 (6.0)	0.015
**FACTORS AFFECTING HEALTH**			
History of coded normal general examination	13371 (32.2)	12981 (31.3)	0.02
History of coded abnormal general examination	248 (0.6)	200 (0.48)	0.016
History of cancer screening	13869 (33.4)	13375 (32.3)	0.025
Long-term drug therapy	9231 (22.3)	8735 (21.1)	0.029
History of nicotine dependence	4005 (9.7)	3868 (9.3)	0.011
Prior influenza vaccine	41466 (100.0)	41466 (100.0)	NA

Supplementary Table 14 Baseline characteristics for the comparison between live zoster vaccine and Tdap vaccine. SMD=Standardised mean difference.

	Live	Tdap vaccine	SMD
**Number**	64035	64035	-
**SOCIODEMOGRAPHICS**			
Age; mean (SD); y	71.2 (4.9)	71.2 (4.9)	0.00087
Sex; n (%)			
Female	33858 (52.9)	33830 (52.8)	0.00088
Male	28998 (45.3)	29159 (45.5)	0.005
Other/Unknown	1179 (1.8)	1046 (1.6)	0.016
Race; n (%)			
White	47215 (73.7)	47335 (73.9)	0.0043
Black or African American	7135 (11.1)	7412 (11.6)	0.014
Asian	2250 (3.5)	2213 (3.5)	0.0032
Other	1387 (2.2)	1365 (2.1)	0.0024
Unknown	5633 (8.8)	5304 (8.3)	0.018
Ethnicity; n (%)			
Hispanic or Latino	3005 (4.7)	2872 (4.5)	0.0099
Not Hispanic of Latino	50248 (78.5)	50851 (79.4)	0.023
Unknown	10782 (16.8)	10312 (16.1)	0.02
Marital status; n (%)			
Married	20385 (31.8)	20126 (31.4)	0.0087
Never married	4252 (6.6)	4113 (6.4)	0.0088
Divorced	3687 (5.8)	3676 (5.7)	0.00074
Domestic partner	45 (0.07)	51 (0.08)	0.0034
**COMORBIDITIES [ICD-10 code]; n (%)**			
Infections and parasites [A00-B99]	13116 (20.5)	12668 (19.8)	0.017
Herpes simplex infection [B00]	751 (1.2)	732 (1.1)	0.0028
Herpes zoster infection [B02]	1972 (3.1)	1777 (2.8)	0.018
Neoplasms (benign and malignant) [C00-D49]	19816 (30.9)	19218 (30.0)	0.02
Skin cancer [C43-44]	3220 (5.0)	3157 (4.9)	0.0045
In situ neoplasms [D00-09]	1499 (2.3)	1512 (2.4)	0.0013
Benign neoplasms [D10-36]	12045 (18.8)	11506 (18.0)	0.022
Neoplasms of uncertain behaviour [D37-38]	4745 (7.4)	4672 (7.3)	0.0044
Haematological conditions [D50-89]	12754 (19.9)	12588 (19.7)	0.0065
Thyroid disorders [E00-07]	10396 (16.2)	10082 (15.7)	0.013
Iodine-deficiency disorder [E01]	1266 (2.0)	1105 (1.7)	0.019
Hypothyroidism [E03]	8122 (12.7)	7906 (12.3)	0.01
Nontoxic goiter [E04]	2355 (3.7)	2285 (3.6)	0.0059
Hyperthyroidism [E05]	1038 (1.6)	989 (1.5)	0.0061
Other disorders of thyroid [E07]	1321 (2.1)	1202 (1.9)	0.013
Diabetes mellitus [E08-13]	13870 (21.7)	13342 (20.8)	0.02
Vitamin B deficiency [E53]	1876 (2.9)	1849 (2.9)	0.0025

Vitamin D deficiency [E55]	8131 (12.7)	7799 (12.2)	0.016
Overweight and obesity [E66]	8729 (13.6)	8251 (12.9)	0.022
Metabolic disorders [E70-88]	33132 (51.7)	31728 (49.5)	0.044
Psychiatric comorbidities			
Substance use disorder [F10-19]	6385 (10.0)	6067 (9.5)	0.017
Mood disorder [F30-39]	9093 (14.2)	8696 (13.6)	0.018
Anxiety disorder [F40-48]	8837 (13.8)	8504 (13.3)	0.015
Organic disorder [F50-59]	1620 (2.5)	1620 (2.5)	0
Neurological disorder [G00-99]	23185 (36.2)	22413 (35.0)	0.025
Ophthalmic disorder [H00-59]	16533 (25.8)	15951 (24.9)	0.021
Ear/mastoid process disease [H60-95]	11438 (17.9)	10901 (17.0)	0.022
Cardiovascular disease [I00-99]	38484 (60.1)	36978 (57.7)	0.048
Chronic rheumatic heart diseases [I05-09]	1784 (2.8)	1721 (2.7)	0.006
Hypertension [I10]	32430 (50.6)	31033 (48.5)	0.044
Ischaemic heart disease [I20-25]	10120 (15.8)	9851 (15.4)	0.012
Other heart disease [I30-52]	14834 (23.2)	14468 (22.6)	0.014
Cerebrovascular diseases [I60-69]	5561 (8.7)	5332 (8.3)	0.013
Cerebral infarction [I63]	2031 (3.2)	1995 (3.1)	0.0032
Atherosclerosis [I70]	2890 (4.5)	2828 (4.4)	0.0047
Other peripheral vascular diseases [I73]	3130 (4.9)	3042 (4.8)	0.0064
Other disorders of arteries/arterioles [I77]	1646 (2.6)	1608 (2.5)	0.0038
Disease of veins/lymphatics [I80-89]	5204 (8.1)	5170 (8.1)	0.0019
Unspecified disease of circulatory system [I95-99]	3633 (5.7)	3600 (5.6)	0.0022
Respiratory disease [J00-99]	24443 (38.2)	23463 (36.6)	0.032
Digestive disease [K00-95]	27708 (43.3)	26777 (41.8)	0.029
Dermatological disease [L00-99]	20247 (31.6)	19621 (30.6)	0.021
Musculoskeletal disease [M00-99]	36230 (56.6)	34818 (54.4)	0.044
Genitourinary disease [N00-99]	26119 (40.8)	25285 (39.5)	0.027
Congenital malformation [Q00-99]	3880 (6.1)	3753 (5.9)	0.0084
Injury/poisoning [S00-T88]	22846 (35.7)	21005 (32.8)	0.061
Falls [W00-19]	4162 (6.5)	3241 (5.1)	0.062
**FACTORS AFFECTING HEALTH**			
History of coded normal general examination	17453 (27.3)	15906 (24.8)	0.055
History of coded abnormal general examination	300 (0.47)	275 (0.43)	0.0058
History of cancer screening	17509 (27.3)	16140 (25.2)	0.049
Long-term drug therapy	11997 (18.7)	11497 (18.0)	0.02
History of nicotine dependence	5216 (8.1)	4931 (7.7)	0.016
Prior influenza vaccine	23845 (37.2)	24466 (38.2)	0.02

References

1. Taquet, M. *et al.* Incidence, co-occurrence, and evolution of long-COVID features: A 6-month retrospective cohort study of 273,618 survivors of COVID-19. *PLoS Med.* **18**, e1003773 (2021).
2. Casey, J. A., Schwartz, B. S., Stewart, W. F. & Adler, N. E. Using Electronic Health Records for Population Health Research: A Review of Methods and Applications. *Annu. Rev. Public Health* **37**, 61–81 (2016).
3. Cowie, M. R. *et al.* Electronic health records to facilitate clinical research. *Clin. Res. Cardiol.* **106**, 1–9 (2017).
4. Jetley, G. & Zhang, H. Electronic health records in IS research: Quality issues, essential thresholds and remedial actions. *Decis. Support Syst.* **126**, 113137 (2019).
5. Hsu, C.-C. *et al.* Association of dementia and peptic ulcer disease: A nationwide population-based study. *Am. J. Alzheimers. Dis. Other Demen.* **31**, 389–394 (2016).
6. Li, X., Huang, L., Tang, Y., Hu, X. & Wen, C. Gout and risk of dementia, Alzheimer's disease or vascular dementia: a meta-epidemiology study. *Front. Aging Neurosci.* **15**, 1051809 (2023).
7. Iacus, S. M., King, G. & Porro, G. Causal inference without balance checking: Coarsened Exact Matching. *Polit. Anal.* **20**, 1–24 (2012).
