# Supplementary material for: The recombinant shingles vaccine is associated with lower risk of dementia
Source: Nat Med. 2024 Jul 25;30(10):2777–81. doi: 10.1038/s41591-024-03201-5 (PMC11485228; doi:10.1038/s41591-024-03201-5)
Supplement: Supplementary file 2 — Reporting Summary [file 41591_2024_3201_MOESM2_ESM.pdf]

nature portfolio

Corresponding author(s): Maxime Taquet and Paul J Harrison

Last updated by author(s): 2024/03/13

Reporting Summary

Nature Portfolio wishes to improve the reproducibility of the work that we publish. This form provides structure for consistency and transparency in reporting. For further information on Nature Portfolio policies, see our Editorial Policies and the Editorial Policy Checklist.

Statistics

For all statistical analyses, confirm that the following items are present in the figure legend, table legend, main text, or Methods section.

n/a	Confirmed	
☐	☒	The exact sample size (*n*) for each experimental group/condition, given as a discrete number and unit of measurement
☐	☒	A statement on whether measurements were taken from distinct samples or whether the same sample was measured repeatedly
☐	☒	The statistical test(s) used AND whether they are one- or two-sided<br>*Only common tests should be described solely by name; describe more complex techniques in the Methods section.*
☐	☒	A description of all covariates tested
☐	☒	A description of any assumptions or corrections, such as tests of normality and adjustment for multiple comparisons
☐	☒	A full description of the statistical parameters including central tendency (e.g. means) or other basic estimates (e.g. regression coefficient) AND variation (e.g. standard deviation) or associated estimates of uncertainty (e.g. confidence intervals)
☐	☒	For null hypothesis testing, the test statistic (e.g. *F*, *t*, *r*) with confidence intervals, effect sizes, degrees of freedom and *P* value noted<br>*Give P values as exact values whenever suitable.*
☒	☐	For Bayesian analysis, information on the choice of priors and Markov chain Monte Carlo settings
☒	☐	For hierarchical and complex designs, identification of the appropriate level for tests and full reporting of outcomes
☐	☒	Estimates of effect sizes (e.g. Cohen's *d*, Pearson's *r*), indicating how they were calculated

Our web collection on statistics for biologists contains articles on many of the points above.

Software and code

Policy information about availability of computer code

Data collection	Data collection is performed using TriNetX user interface. Demographics are coded to HL7 version 3 administrative standards, diagnoses are represented by ICD-10-CM codes, procedures are coded in ICD-10-PCS or CPT, and measurements are coded to LOINC.
Data analysis	Propensity-score matching was achieved within TriNetX by calculating the propensity score with the scikit-learn package in Python 3.7 and using a nearest neighbour search with numpy 1.21.5 in Python 3.7. Data analysis includes all the processing of data from the life tables of matched cohorts and include Kaplan-Meier estimation, restricted mean time lost estimates, bootstrapping, permutation test and generation of figures. All these steps were conducted in R version 4.2.1. The assumption that the hazards were proportional was tested using the generalized Schoenfeld approach implemented in the cox.zph function of the survival package (version 3.2.3). The restricted mean time lost was calculated using R package survRM2 version 1.0.4. Bootstrapping and permutation tests were conducted using in-house R code that is publicly available. Time-varying hazard ratios were calculated using the generalized survival models of the rstpm2 package (version 1.5.1) in R.<br><br>All in-house R code is available via the following link: https://osf.io/9frxm/

For manuscripts utilizing custom algorithms or software that are central to the research but not yet described in published literature, software must be made available to editors and reviewers. We strongly encourage code deposition in a community repository (e.g. GitHub). See the Nature Portfolio guidelines for submitting code & software for further information.

Data

Policy information about availability of data

All manuscripts must include a data availability statement. This statement should provide the following information, where applicable:

- Accession codes, unique identifiers, or web links for publicly available datasets
- A description of any restrictions on data availability
- For clinical datasets or third party data, please ensure that the statement adheres to our policy

The TriNetX system returned the results of these analyses as csv files, which we downloaded and archived. Aggregate data, as presented in this article, can be freely accessed at https://osf.io/9frxm/. The data used for this article were acquired from TriNetX. This study had no special privileges. Inclusion criteria specified in the Methods would allow other researchers to identify similar cohorts of patients as we used here for these analyses; however, TriNetX is a live platform with new data being added daily so exact counts will vary. To gain access to the data, a request can be made to TriNetX (join@trinetx.com), but costs might be incurred, and a data sharing agreement would be necessary.

Research involving human participants, their data, or biological material

Policy information about studies with human participants or human data. See also policy information about sex, gender (identity/presentation), and sexual orientation and race, ethnicity and racism.

Reporting on sex and gender	Analysis stratified by sex was performed. The sex was as recorded in the individual's electronic health record.
Reporting on race, ethnicity, or other socially relevant groupings	Self-reported race and ethnicity in TriNetX come from the individual's electronic health record. TriNetX maps race to the following categories: Asian, American Indian or Alaskan Native, Black or African American, Native Hawaiian or Other, White, Unknown race. It maps ethnicity to the following categories: Hispanic or Latino, Not hispanic or latino, Unknown ethnicity.
Population characteristics	All these characteristics are reported in full in Table 1 and Supplementary Tables 2-9.
Recruitment	There was no recruitment as this was a real-world analysis based on electronic health records data. All individuals with a recorded shingles vaccine at the age of 65 or over, between October 2014 and October 2020 and who did not have a diagnosis of a neurodegenerative condition before their vaccine were included.
Ethics oversight	De-identification data are formally attested as per Section §164.514(b)(1) of the HIPAA Privacy Rule, superseding TriNetX's waiver from the Western Institutional Review Board; no further ethical approval was thus needed.

Note that full information on the approval of the study protocol must also be provided in the manuscript.

Field-specific reporting

Please select the one below that is the best fit for your research. If you are not sure, read the appropriate sections before making your selection.

☒ Life sciences ☐ Behavioural & social sciences ☐ Ecological, evolutionary & environmental sciences

For a reference copy of the document with all sections, see nature.com/documents/nr-reporting-summary-flat.pdf

Life sciences study design

All studies must disclose on these points even when the disclosure is negative.

Sample size	This is a real-world data analysis which includes all individuals who received a shingles vaccine between October 2014 and October 2020. No sample size calculation was therefore performed.
Data exclusions	Pre-established exclusion criteria included any pre-existing neurodegenerative condition before shingles vaccination. These include dementia (since individuals could not have the outcome before the exposure) and other neurodegenerative conditions (such as Parkinson's disease) since these might indicate that a disease process that might lead to to dementia has already started.
Replication	Findings were replicated in 9 scenarios: (i) when restricting cohorts to those who received the predominant vaccine; (ii) when restricting exposure windows to 6 months either side of the step change; (iii) using coarsened exact matching; after aligning follow-up times (iv) at the cohort level and (v) the individual level; (vi) after adjusting for socioeconomic deprivation; (vii) among women and (viii) men; and (ix) after limiting follow-up so that it is fully contained before the COVID-19 pandemic. All attempts at replication were successful.
Randomization	The influence of covariates was strongly limited by the use of a natural experiment created by the rapid transition from the use of live shingles vaccine to the use of recombinant shingles vaccine. Remaining covariates imbalance (e.g. due to drift in characteristics of the population being vaccinated) were further controlled for by propensity-score matching (in the primary analysis) and by coarsened exact matching (in a robustness analysis).
Blinding	Cohorts were created based on predefined inclusion and exclusion criteria. The outcome was ascertained based on routine electronic health records data and not by any investigator of this study. As a result, blinding was not necessary.

Reporting for specific materials, systems and methods

We require information from authors about some types of materials, experimental systems and methods used in many studies. Here, indicate whether each material, system or method listed is relevant to your study. If you are not sure if a list item applies to your research, read the appropriate section before selecting a response.

Materials & experimental systems

n/a	Involved in the study
☒	☐ Antibodies
☒	☐ Eukaryotic cell lines
☒	☐ Palaeontology and archaeology
☒	☐ Animals and other organisms
☒	☐ Clinical data
☒	☐ Dual use research of concern
☒	☐ Plants

Methods

n/a	Involved in the study
☒	☐ ChIP-seq
☒	☐ Flow cytometry
☒	☐ MRI-based neuroimaging

Plants

Seed stocks	N/A
Novel plant genotypes	N/A
Authentication	N/A
